# The hazardous 2017–2019 surge and river damming by Shispare Glacier, Karakoram

**DOI:** 10.1038/s41598-020-61277-8

**Published:** 2020-03-13

**Authors:** Rakesh Bhambri, C. Scott Watson, Kenneth Hewitt, Umesh K. Haritashya, Jeffrey S. Kargel, Arjun Pratap Shahi, Pritam Chand, Amit Kumar, Akshaya Verma, Himanshu Govil

**Affiliations:** 1 Centre for Glaciology, Wadia Institute of Himalayan Geology, 33 GMS Road, Dehradun, 248001 India; 2 COMET, School of Earth and Environment, University of Leeds, Leeds, UK; 3 Department of Hydrology & Atmospheric Sciences, University of Arizona, Arizona, USA; 4 Department of Geography and Environmental Studies, Wilfrid Laurier University, Waterloo, Canada; 5 Department of Geology, University of Dayton, 300 College Park, Dayton, OH 45469 USA; 6 Planetary Science Institute, Tucson, AZ 85719 USA; 7 Department of Applied Geology, National Institute of Technology, Raipur, Chhattisgarh 492010 India; 8 Department of Geography, Central University of Punjab, Bathinda, India

**Keywords:** Cryospheric science, Environmental sciences

## Abstract

In 2017–2019 a surge of Shispare Glacier, a former tributary of the once larger Hasanabad Glacier (Hunza region), dammed the proglacial river of Muchuhar Glacier, which formed an ice-dammed lake and generated a small Glacial Lake Outburst Flood (GLOF). Surge movement produced the highest recorded Karakoram glacier surface flow rate using feature tracking (~18 ± 0.5 m d^−1^) and resulted in a glacier frontal advance of 1495 ± 47 m. The surge speed was less than reports of earlier Hasanabad advances during 1892/93 (9.3 km) and 1903 (9.7 km). Surges also occurred in 1973 and 2000–2001. Recent surges and lake evolution are examined using feature tracking in satellite images (1990–2019), DEM differencing (1973–2019), and thermal satellite data (2000–2019). The recent active phase of Shispare surge began in April 2018, showed two surface flow maxima in June 2018 and May 2019, and terminated following a GLOF on 22–23 June 2019. The surge likely had hydrological controls influenced in winter by compromised subglacial flow and low meltwater production. It terminated during summer probably because increased meltwater restored efficient channelized flow. We also identify considerable heterogeneity of movement, including spring/summer accelerations.

## Introduction

**Figure 1:**
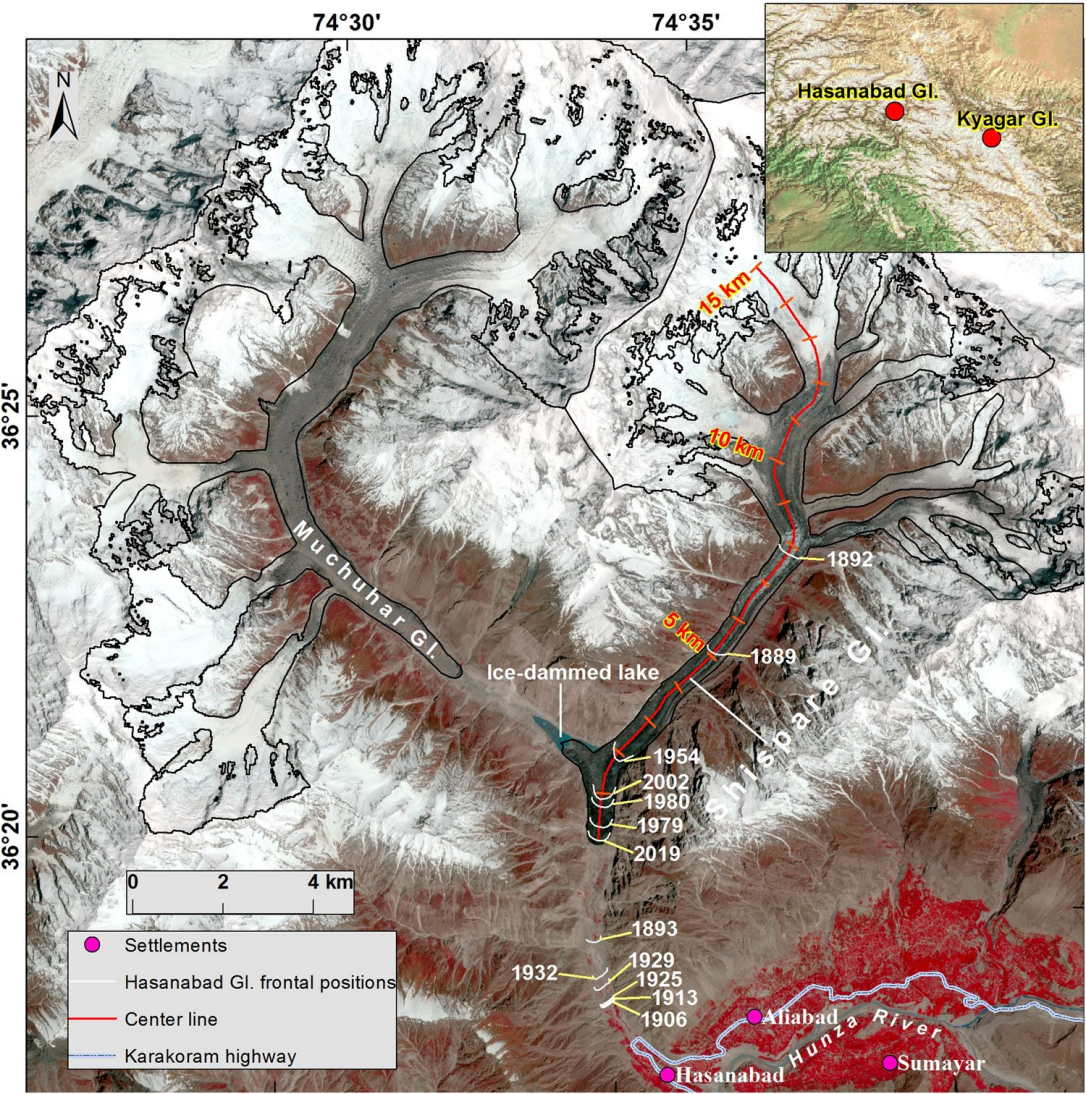
Overview of the Hasanabad Glacier (= formerly coalescent, now detached Shispare and Muchuhar tributaries). Shispare and Muchuhar glaciers advanced to within ~7.0 and ~12.0 km from the Hunza River, respectively, by the time of this 31 May 2019 Sentinel-2 NIR-RED-BLUE image. The center line is used for elevation change (15 km up-glacier from terminus) and surface displacement (12 km up-glacier from terminus) extraction. The terminus of Hasanabad Glacier between 1889 and 2019 is based on Supplementary Table 1 (modified from Goudie *et al*.^36^). The glacier outline was modified from the Randolph Glacier Inventory (RGI 6.0)^58^.

Hasanabad was a surge-type glacier situated on the north flank of Hunza Valley in the Central Karakoram (Fig. 1). During most, but not all, of the 20th Century, the Shispare (or Shisper) and Muchuhar tributaries of the Hasanabad Glacier have been separated. Shispare Glacier recently gained attention of the media, scientific community, policy makers, and disaster response agencies when it surged, blocked the outlet stream of Muchuhar Glacier and formed a lake^1–5^ (referred to hereafter as Shispare Lake), which then drained, and has recently begun reforming.

Surge-type glaciers oscillate between brief (months to years) rapid flow and lengthy (tens to hundreds of years) slow flow or stagnation, which are called the ‘active’ (or ‘surge’) and ‘quiescent’ phases, respectively^6^. In the former, a large volume of the glacier’s mass is rapidly transferred from an upper ‘reservoir zone’ into the lower ‘receiving zone.’ Conversely, in the quiescent phase, the lower glacier slowly stagnates, thins, and retreats, while the upper part builds mass towards another surge^6,7^. Surging occurs in both temperate and polythermal glaciers^6–11^. Earlier, two primary mechanisms were proposed to explain the change between surge and quiescent flow. (1) Thermally regulated surges generally switch from largely cold-based ice in quiescence, to warm-based ice during the surge (e.g., Monacobreen Glacier, Svalbard)^12^. (2) Hydrological regulation (e.g., Variegated Glacier, Alaska) invokes switching from low water pressure in subglacial channels of temperate glaciers to high water pressure, usually with restructuring of subglacial drainage^8,11,13,14^. However, more recently, a uniform model of surging has been proposed to integrate both processes under an enthalpy framework^7^. The enthalpy balance theory explains thermally and hydrological regulated surges in a single model; the surges occur due to enthalpy gains over losses, and subsequent build-up of water at the bed^15,16^.

Glacier surges were first reported for the Karakoram during the 19^th^ century^17^. At least 220 glaciers are identified as surge-type and represent >40% of the Karakoram glacierized area^18–20^. Active surges pose dangerous hazards for local communities, threatening destruction of critical infrastructure when ice overrides villages, roads, bridges, and trails. Furthermore, if the glacier advances across river valleys and forms an ice-dammed lake, it may result in devastating outburst floods^21–24^. Therefore, monitoring of glacier surges, ice-dammed lake formation and drainage is essential.

In the Karakoram, a large surge-like behavioral spectrum of surface movement has been reported, but the processes controlling their evolution may differ on a glacier-by-glacier basis^20,24–27^. The hydrological surges, referred to as classic surges in the literature^28–30^ involve sudden onset with high maximum velocities, and low velocities following a rapid termination of the surge. Classic surges involve ten- to twenty-fold accelerations of ice movement. However, some recent work documents many cases of lesser, two- to three-fold, accelerations coupled with the longer ‘quiescent phase’ and leading to recognition of a spectrum, or different types, of unsteady or heterogeneous glacier motions^24,26,29^. Unsteady glacier flow complicates glaciologists’ ability to make accurate assessments of individual glacier mass balances using *in-situ* observations because of the uncertain outcomes or significance of surge phase and quiescent phase flow.

The subglacial processes and conditions are key (e.g., amount of debris, distribution of stored water and temperature gradient)^9,31,32^ to understand the diversity of surge-types and surge-like behavioral spectrum. However, such information is rare or unknown in the Karakoram, because ground-based observations are difficult to acquire. In the absence of field observations, multi-temporal glacier surface displacement and elevation change data from multiple satellite images and DEMs offer invaluable evidence towards understanding surge mechanisms. Recent advances in high-resolution remote sensing satellite data and DEMs have shown great potential to track and assess changes in glacier surface displacement, mass transfer from ‘reservoir’ to ‘receiving’ zones, and fluctuations in surface melt elevation. Several studies in the Karakoram have utilized remote sensing data to study glacier surge dynamics^19,20,23–27,33,34^. More recently, Rashid *et al*.^35^ reported the recent surge of Shispare Glacier using remote sensing observations. This study mainly presented monthly changes in the surface displacement of Shispare Glacier using only Landsat 8 OLI data. However, due to cloud and snow conditions, this data source alone may fail to quantify rapid acceleration and deceleration of surface displacement^20,24^, which prove typical in the surge mechanism of Karakoram glaciers. Therefore, the aims of our study are (1) to improve the understanding of Shispare Glacier surge dynamics and (2) to compare with the dynamics of other glacier surges. We accomplish the first aim with the use of multi-temporal and multi-sensor satellite images (Landsat 8 OLI, ASTER and Sentinel-2), digital elevation models (DEMs) of difference, and satellite thermal data to assess surface melt.

## Study site, surge and lake history

Shispare (36.40°N 74.61°E) and Muchuhar (36.43°N 74.50°E) glaciers, former tributaries of the once larger Hasanabad Glacier, are situated in Hunza Valley, Gilgit-Baltistan, Pakistan. Shispare Glacier covers 53.7 km^2^ at an elevation range of 2567–7611 m a.s.l. The Hasanabad Glacier is noted, historically (1892–3), for claims that it had the longest and fastest terminus advance (9.3 km) of any known Karakoram glacier^17,29,36,37^. During 1903, it had another terminus advance of nearly equal magnitude (9.7 km advance in 2.5 months)^38^ (Supplementary Table S1). If true, that would mean an average speed of 130 m d^−1^, which would be the fastest documented surge in the world. According to Jiskoot^6^ the most rapid glacier surge known globally was 125 m d^−1^ or 10 km yr^−1^ for Bruarjokull in Iceland (p. 417). Mason^17^ noted evidence available then for Hasanabad that supported “…the reputation of having undergone greater fluctuations than any other glacier in the world” (p. 232). However, reports from the 1980s preceded recognition of surge-type glaciers and the present understanding of surge dynamics. Nevertheless, recent events remove certain doubts about those two separate, rapid and exceptional advances, and support the orders of magnitude of accelerated flow suggested in historical reports. Moreover, it now seems clear the paradox of two huge advances within a decade is because each of Hasanabad’s main tributaries, Shispare and Muchuhar, are surge-type and, as is generally true for surge-type tributaries, have independent surge-cycles^39^. The present (2017–2019) Hasanabad surge is of the Shispare branch^24^. Assuming the surge intervals have remained approximately the same, a surge from Muchuhar can be expected later in the century, possibly within a decade or so.

In the 20^th^ century, ground surveys and maps suggested that the two tributaries Shispare and Muchuhar retreated and had separated into distinct glaciers by 1954^40^. However, because of the surge of Muchuhar, both tributaries met again before 1972, although the main terminus retreated ~4 km from 1979–2013^20^. It has not again connected to Shispare to date^20^. Small surges of Shispare Glacier occurred between 1972–76 and 1993–2002^20,24^. Then, in 2017–2018, a major surge of this branch was reported^24^. It destroyed an irrigation channel, section of an under-construction hydroelectric plant, shepherds’ trails, and access to summer pastures^3–5^. At least five minor damaging GLOFs associated with surges have been documented between 1890 and 1905 at Hasanabad Glacier^41^.

## Results

We detected three surges (1973; 2000–2001; 2017–2019) using satellite images from 1973 to 2019. However, the ice dam of Shispare Glacier formed a lake only in the most recent surge due to blockage of the Muchuhar outlet stream. This suggests that the recent surge was more extensive than the previous two. In the following sections, we present results of surface displacement, elevation change, surface melt elevations, frontal advance, lake formation and drainage.

### Surface displacement and frontal change

For Shispare Glacier, we computed 70 surface displacement vector fields between 1990 and 2019 using CIAS^42^, and 42 measurements using COSI-Corr^43^ (2016–2019) to cross-reference the results (see Supplementary Tables 2 and 3 for image details and uncertainties). In the last three decades, two surges and several accelerations and decelerations in surface displacements were observed. During the period 1990–1996, surface displacements were low (<1 m d^−1^) across the glacier (Supplementary Fig. S1a). Between 23 July 1999 and 06 May 2000, ~1.5 km up-glacier from the terminus, a surge front was observed and surface displacements were >1 m d^−1^ (Supplementary Fig. S1b). Between early May 2000 and July 2000, the surge front advanced 1 km down valley, reaching the terminus with a speed of 2 ± 0.1 m d^−1^ (Supplementary Fig. S1b). Thereafter, surface displacements decreased and, from 15 July 2002 to 31 May 2003, were on average ~0.3 m d^−1^ over the whole glacier surface (Supplementary Fig. S1c). Based on ASTER images from 2004 to 2011, maximum surface displacement fluctuated between ~0.4 m d^−1^ to ~0.6 m d^−1^ (Supplementary Fig. S2b,c). Seasonal changes from 1990 to 2011 could not be computed due to cloud cover and seasonal snowfall on the glacier.

**Figure 2:**
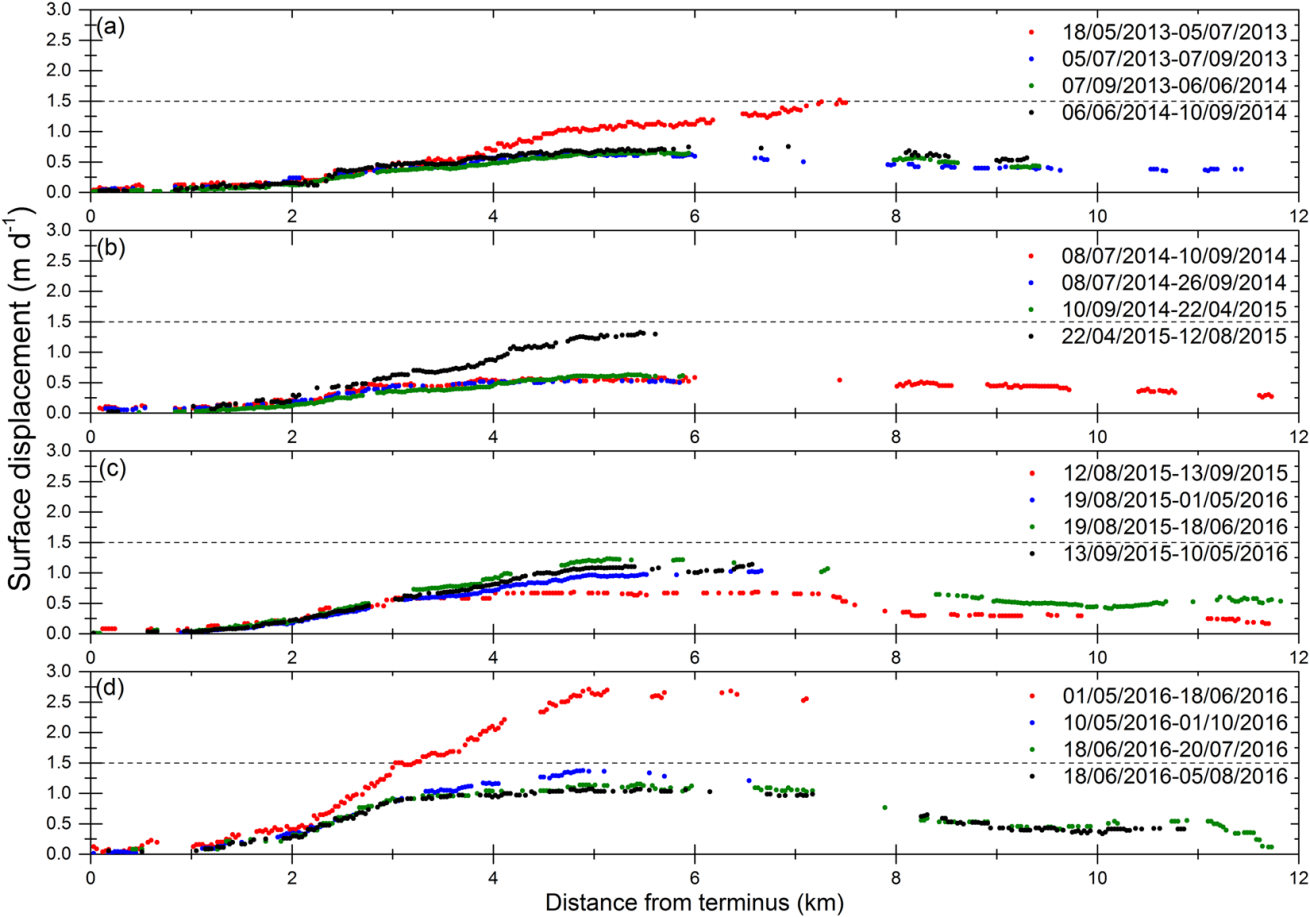
CIAS-derived surface displacement of Shispare Glacier during 2013–2016.

On many occasions between 2013 and 2016, in early summer months (April to June), we observed two- to three-fold accelerations in surface displacement (Fig. 2a–d). The highest velocity (3 ± 0.1 m d^−1^) was observed between 01 May 2016 and 18 June 2016, but no evidence of a surge was observed near the terminus (Fig. 2d). It is not unusual in the Karakoram to detect acceleration and deceleration of surge glaciers during the quiescent phase^20,24^. Between mid-June and through August 2016, the surface displacement suddenly decreased by more than half (Fig. 2d). However, comprehensive analysis for summer months of 2017 was incomplete because of seasonal snow and cloud cover in Landsat 8 OLI and Sentinel-2 images. We observed slight acceleration in surface displacement in May-June 2017 compared to previous months but less than the May-June 2016 velocity. An uncertainty arises because the end of the month (May 29^th^) image was utilized for 2017, whereas the beginning of the month (May 1^st^) image was used for 2016, and the expected seasonal change may partly give an impression of interannual acceleration.

The pre-surge acceleration of Shispare Glacier was observed in winter months when surface displacements doubled between November 2017 (1 ± 0.1m d^−1^) and February 2018 (2 ± 0.1 m d^−1^) (Supplementary Fig. S3c). During this period, the spatial pattern of surface acceleration provided no evidence of a surge wave moving down the glacier, unlike some other glaciers in the region^20,26^. The maximum acceleration occurred between 4 km and 7 km up-glacier from the terminus. No change was observed in the terminus position. The fluctuations in surface displacement during 2013 to 2016 (acceleration and deceleration) mask or preclude a lengthy pre-surge acceleration as observed on other surge-type glaciers^6,22,26,44^ (Fig. 2a–d). The pre-surge acceleration duration for Shispare Glacier from November 2017 to February 2018 appears rather brief compared to other surges reported in the Karakoram^22^.

**Figure 3:**
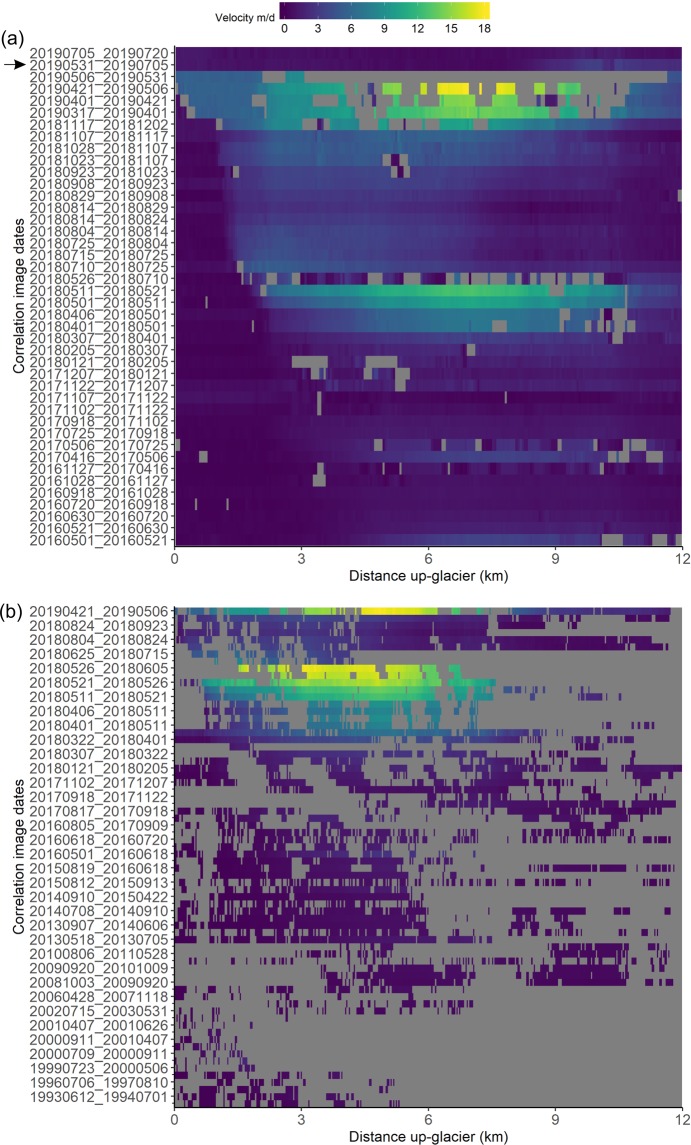
Surface displacement of Shispare Glacier: (**a**) 2016–2019 computed using COSI-Corr software and (**b**) 1990–2019 computed using CIAS software. Every other tick mark is labeled in (**b**). See Supplementary Tables 2 and 3 for the complete list of correlations. The black arrow on (**a**) indicated when the lake drainage occurred (22–23 June 2019).

For Shispare, the active phase could be detected in April 2018 when maximum surface displacement reached ~6 ± 0.1 m d^−1^ between 06 April 2018 and 11 May 2018. At the beginning of June, peak surge displacement increased suddenly to ~18 ± 0.5 m d^−1^ (Supplementary Fig. S4b). The surge of Shispare Glacier caused a three-fold acceleration from April to May 2018 and approximately a 20-fold acceleration since September 2017 (Fig. 3). However, then there was a sudden deceleration until mid-September 2018 (Supplementary Fig. S4d) and surface displacements reduced to ~2.5 m d^−1^ (Supplementary Fig. S5). The surface displacement again slightly accelerated to ~3.5 m d^−1^ in October 2018. During October 2018 to April 2019, we could not get consistent surface displacement data from the CIAS software (Fig. 3b). However, COSI-Corr performed better than CIAS during this period, during which surface displacement increased three-fold. There followed a new phase with a maximum flow velocity (surge peak) (~18 ± 0.3 m d^−1^) between 21 April 2019 and 06 May 2019 (Fig. 3; Supplementary Fig. S6b). The speed of surge peaks in 2018 and 2019 was similar (~18 ± 0.3 m d^−1^), but the 2019 surge peak occurred at the end of April whereas the 2018 surge peak occurred at the end of May. A large contrast in the timings of two surge peaks has also been reported for other surge-type glaciers (e.g., Kyagar, Hispar) in the Karakoram^22,27^ and Alaska (e.g., Bering Glacier)^45^. For instance, two surge speed maxima of Kyagar Glacier occurred in September 2014 and July 2015. Between 06 May 2019 and 31 May 2019, surface displacement of Shispare Glacier abruptly decreased 50% (~9 m d^−1^), then further reduced to ~1 m d^−1^ by mid-July 2019 at 3 km up-glacier from the terminus. Shispare Lake drainage on 22–23 June occurred during the latter period of slowdown (Fig. 3). The loss of the Shispare Lake water may have relieved the hydrostatic pressure and deprived the system of basal water upon which the glacier was sliding^13^. In all, Shispare Glacier’s terminus advanced 1495 ± 47 m between 2017 and 2019. However, the recent advance is about six times less than reports of earlier Hasanabad advance during 1892/93 surge^17,36,37^ (9.3 km) and the 1903 surge (9.7 km)^17,38^.

### Surface elevation change

**Figure 4:**
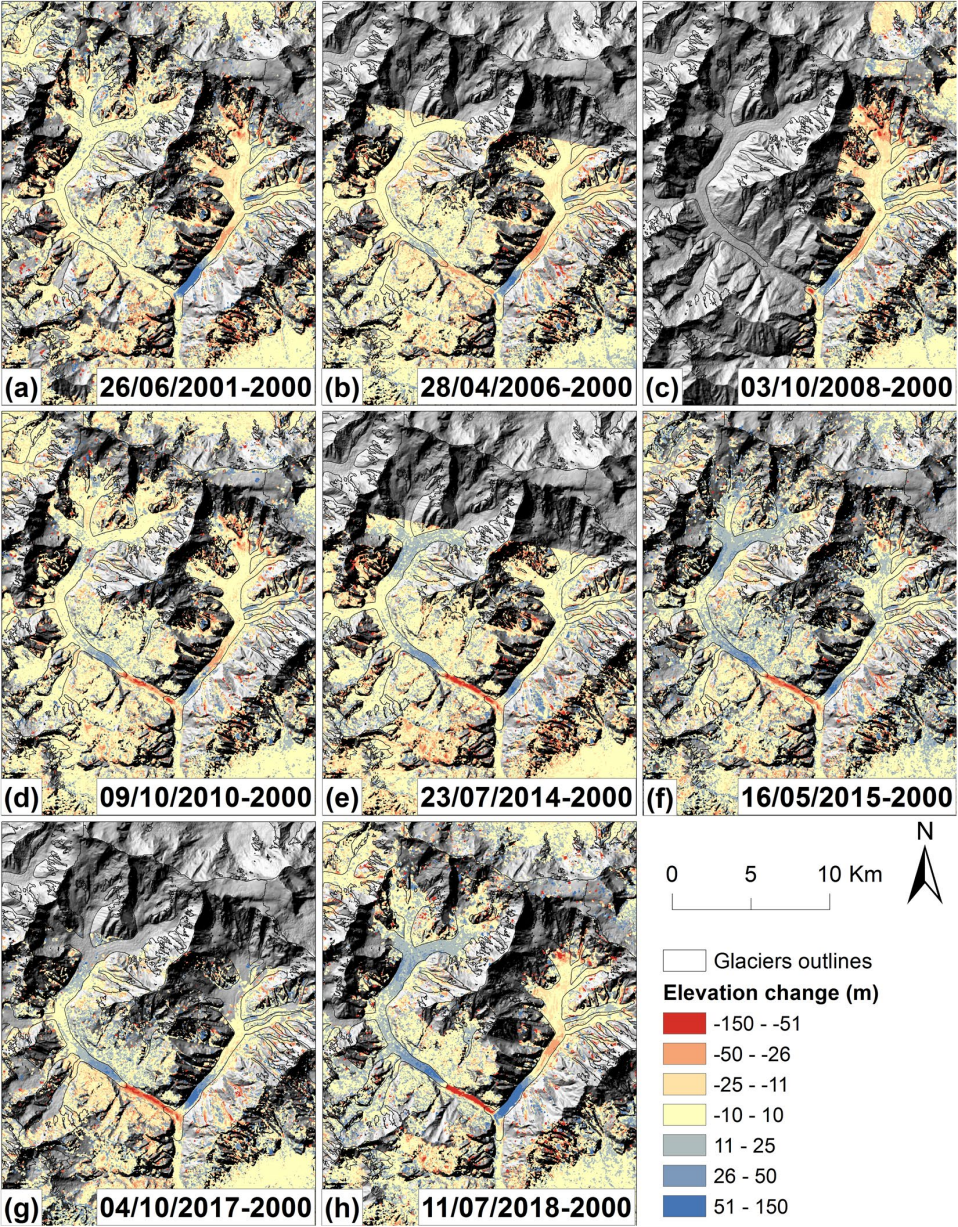
Elevation change of Hasanabad Glacier (Shispare and Muchuhar) using multiple ASTER and SRTM DEMs after coregistration.

**Figure 5:**
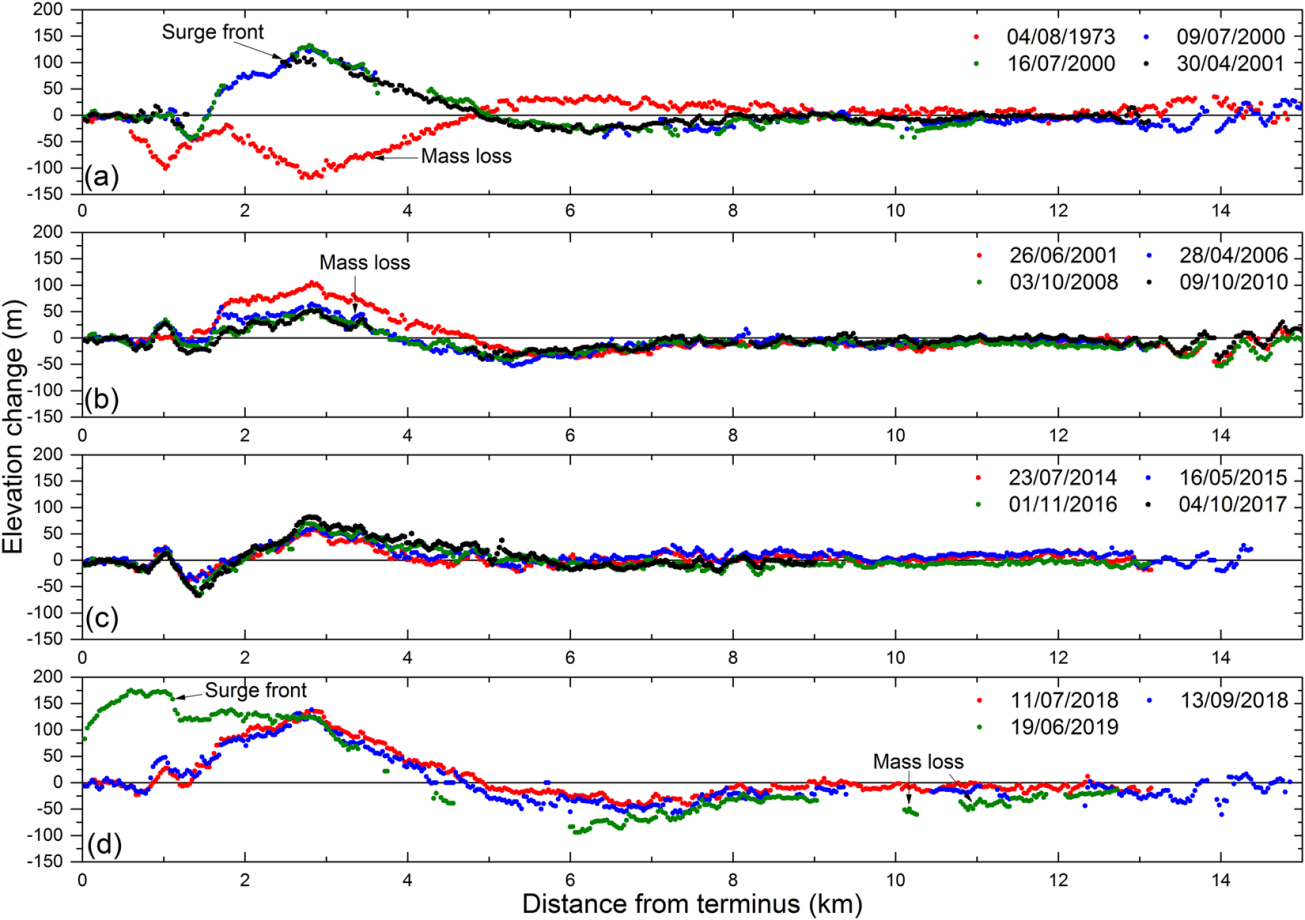
Elevation change of Shispare Glacier using KH-9 Hexagon, multiple ASTER and the SRTM DEMs. The KH-9 Hexagon and ASTER DEMs (dates presented in the legend) were subtracted from the reference SRTM DEM to compute elevation changes.

In total, 14 elevation change maps were generated through the multiple ASTER, KH-9 Hexagon and SRTM DEMs (Figs. 4 and 5; Supplementary Table 4; Supplementary Figs. S7–S9). Previous studies suggest that Shispare Glacier surged in 1972–76 (Supplementary Table 1). However, we could not quantify mass transfer from the reservoir zone to the receiving zone for 1972–76 due to lack of an additional 1970s Hexagon DEM. During 1973 to 2000, the average elevation change in the lower receiving zone (4 km upstream to terminus) was −61 ± 28 m. Our surface displacement data suggest that Shispare Glacier surged in 2000–2001 (Supplementary Fig. S1). During 2001, the average elevation of the lower receiving zone experienced a ~42 ± 20 m gain and the surge front thickened by ~100 ± 20 m (Fig. 5b). Before the recent surge, the lower receiving zone experienced ~28 ± 18 m lowering from 2000–2008 and then remained almost constant until 2016. This observed pattern is a distinctive signature of the quiescent phase, where ice thinned at the lower receiving zone. Most ASTER DEMs failed to map complete elevation information in the reservoir zone due to steep slopes (>40°) and cloud cover. We, therefore, could not reliably quantify elevation changes for the entire reservoir zone. In July 2018, on average, the lower receiving zone rose by 60 ± 21 m compared to 2000 and this thickening was almost doubled (127 ± 21 m) by June 2019. The surge front advanced 2.5 km down valley during this period, reaching the terminus with its thickness of 190 ± 21 m just 600 m up from the terminus (Fig. 5d).

The surface displacements of Shispare Glacier show that the surge was underway by November 2017, but abruptly slowed in June-July 2019, about when the lake drained. We, therefore, considered two ASTER DEMs from 19 June 2019 to 04 October 2017 for ice volume displacement. However, due to voids in the DEMs (mainly because of cloud cover in 19 June 2019 DEM), ice volume displacement was only computed for the receiving zone. The boundary between the reservoir and receiving zones, determined by an elevation change of ±5 m, was observed at 2860 m, 3.5 km from the terminus, where the glacier is 0.6 km wide. However, due to voids in ASTER DEMs we could not find an elevation change of ±0 m to identify the precise boundary between the reservoir and receiving zones, as proposed by Pitte *et al*.^44^. The receiving zone covered 2.2 km^2^ and on average thickened by 114 ± 30 m, resulting in a net gain of 251 ± 67 × 10^6^ m^3^ of ice from June 2019 to October 2017.

### Surface melt elevations

**Figure 6:**
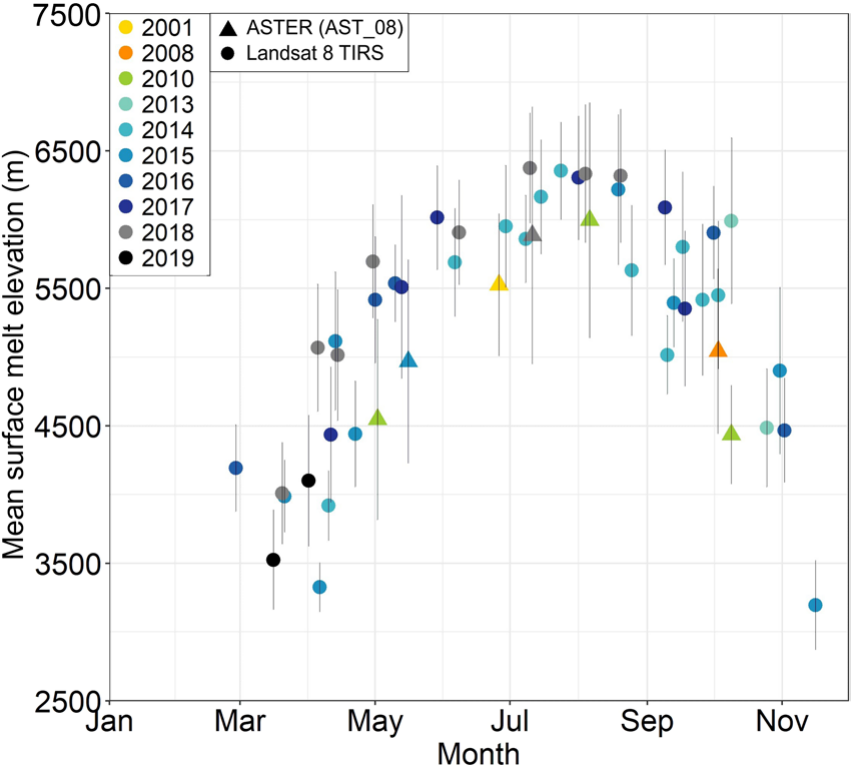
Mean and standard deviation (vertical bars) of surface melt elevations (−1.5C < T < 1.5C) of Shispare Glacier computed using 42 Landsat 8 TIRS (spanning 2013–2019) and 7 ASTER TIR images (spanning 2001–2018).

The surface melt elevations increased from a winter/spring minimum of ~3,500 m to a maximum of ~6,400 m in July/August (Fig. 6). The surface melt elevations derived from ASTER data were lower than Landsat. There was only one near-coincident measurement for comparison. Here, the ASTER surface melt elevation on 11 July 2018 was 5,900 ± 900 m, compared to 6,400 ± 400 m for Landsat on 10 July 2018. We discuss this melt elevation difference in Methods, but in brief, we attribute this difference primarily to a combination of 0.8**°** Celsius bias between ASTER/Landsat, 0.4**°** Celsius difference due to a slightly later time of day of ASTER overpasses compared to Landsat, and differing weather between days and between years of observations.

### Lake formation and drainage

**Figure 7:**
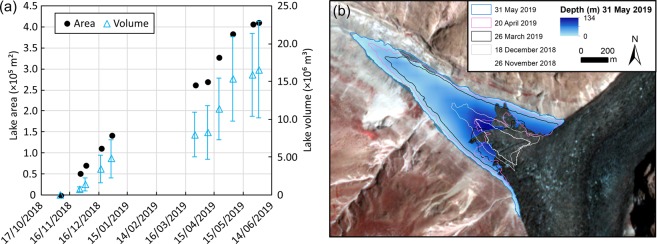
Area and volume time series of the ice-dammed lake.

An ice-dammed Shispare Lake began to form in November 2018 and reached an area of 4.1 × 10^5^ ± 2 × 10^4^ m^2^ and volume of 1.66 × 10^7^ ± 6.4 × 10^6^ m^3^ on 31 May 2019, with a maximum depth of ~134 m (Fig. 7). The glacier advanced into the area occupied by the initial lake and icebergs calved into the lake. Cloud cover restricted satellite image observations over winter and in early June. However, Shispare Lake drained^4^ with a discharge of 142 m^3^/s between 22–23 June 2019, as reported by the Pakistan Meteorological Department. Peak discharge for the glacier dam failure was also estimated using the empirical relationship of Costa and Schuster^46^: where *Q*_*p*_ is the peak discharge and *PE* is the potential energy derived as a product of the dam height (134 m), volume of water (1.66 × 10^7^ m^3^), and the specific weight of water (9,800 N m^−3^). The equation provides a peak discharge of 400 ± 260 m^3^/s. The large uncertainty range is due to a limited number of data points (11) used to derive the relationship but also helps to reflect a wide range of possible outcomes even for similar situations.

**Figure 8:**
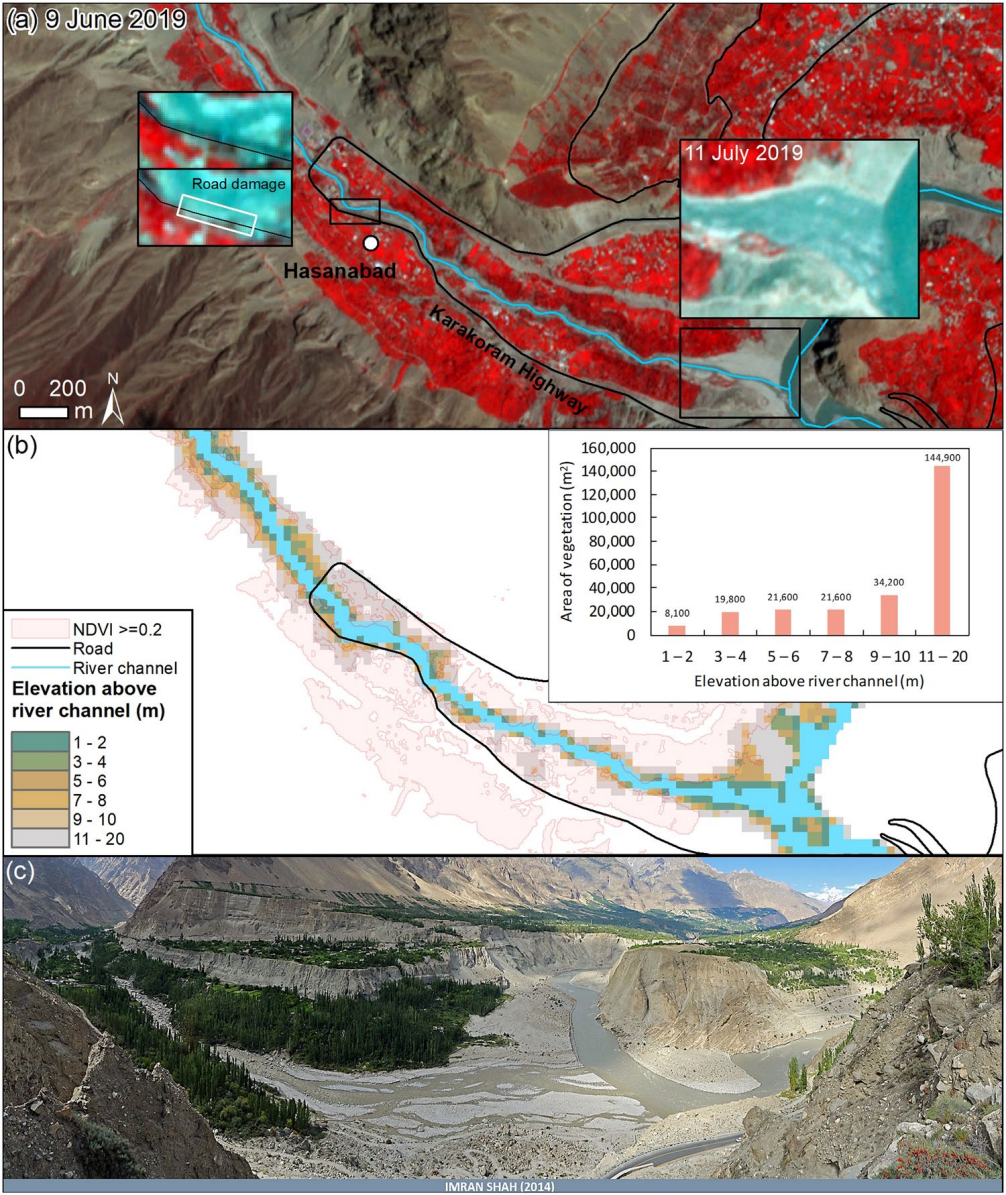
(**a**) PlanetScope image (09 June 2019) of the river channel and adjacent vegetated land (agricultural fields, tree plantations and orchards) before lake drainage, with post-lake drainage insets (11 July 2019)^59^. (**b**) Elevation above the river channel and the area of vegetated land (09 June 2019) within each elevation band. (**c**) Panoramic view in 2014 of the confluence with the Hunza River, overlooking the inset in panel (**a**) (Photograph by Imran Shah. 2014. License: CC BY-SA 2.0).

Comparing PlanetScope images before and after the Shispare Lake drainage showed limited observable vegetation loss attributed to overbank flow, although channel realignment was evident. A ~140 m section of the Karakoram Highway (Fig. 8) appeared to have been affected by flood waters, which was indicated by a colour change suggesting sediment deposition on the road or erosion in this area. The village of Hasanabad and surrounding agricultural land is generally located well above the river channel, giving it some degree of safety, whereas some other developed elements are more prone to GLOF damage.

## Discussion

Earlier studies in Alaska have reported that hydrologically-controlled surges (e.g., Variegated, Bering glaciers) mainly initiate in winter when meltwater is rare or limited and the subglacial flow system is ineffective and distributed (e.g., linked-cavity)^11,13,47^. They usually terminate during the melt season when there is adequate meltwater to restore effective channelised subglacial drainage^13,14^. However, the active surge phase of Shispare Glacier started with the beginning of the 2017 melt season (April–May) and terminated in June–July 2019 (Supplementary Figs. S4 and S6). Similarly, the active surge of Kyagar Glacier, Karakoram began with the onset of the 2014 melt season (April–May) and continued for 15 months (August 2015)^22^. Therefore, the recent surge of Shispare Glacier resembles the hydrologically-controlled surge of Kyagar^22^ but differ from onset timing of Alaskan surge glaciers^13^ (e.g., Variegated, Bering glaciers). We have no field evidence to address the question of why the surge timing of onset is different. The acceleration in glacier flow velocity of Shispare Glacier in successive years of the recent surge can be explained by spring melting of snow (April–June), as the ASTER and Landsat thermal observations (Fig. 6) suggest, and likely penetration of meltwater to the glacier bed^48–51^.

The acceleration and deceleration of glacier flow during the hydrologically-controlled surge is mainly dependent on availability of meltwater at the glacier bed^6,11,13,14,47^. Some water is generated by internal mechanisms (e.g., frictional, pressure and geothermal heat), but most comes from surface meltwater that reaches the bed through crevasses and moulins^14^. The rapid surface velocities of Shispare Glacier during April-June 2018 and April-May 2019 may be attributed to surface meltwater that penetrated to the glacier bed and increased water pressure in an inefficient subglacial drainage system. The shift of surface melt elevations up-glacier from ~3,500 m in winter/spring to ~6,400 m in July/August (summer) indicates continuous but seasonally-variable availability of meltwater (Fig. 6). The intensely crevassed glacier surface, as reported in historical assessment^38^ and surveyed during the recent aerial inspection by helicopter^52^ suggests that these crevasses on glacier surface possibly transferred surface meltwater to the glacial bed. Meltwater significantly lubricates the glacier base in the summer period, which increases basal sliding and the surface movement^53^. However, when meltwater is depleted or hydraulic pressure is reduced due to improved channelized subglacial drainage, basal sliding reduces. Such an evolution of the drainage system during the melt season is hardly explored for Karakoram surge-type glaciers using ground observations, but Alaskan glacier^13^ (e.g., Variegated Glacier) and Icelandic glacier^14^ (e.g., Skeiðarárjökull Glacier) studies have confirmed such mechanisms.

The sudden deceleration of surface movement in June-July 2019, concurrent with the Shispare Lake outburst, exhibits association with subglacial drainage. The subglacial channels possibly opened below the lower receiving zone during the Shispare Lake outburst and re-established an efficient channelized flow that significantly reduced the surface displacement. Such findings for lake outburst and surge termination was reported by Round *et al*.^22^ and Björnsson^14^. The water level of the ice-dammed Shispare Lake depends on supply of meltwater from the Muchuchar Glacier stream and glacier dynamics, geometry, and ice dam stability (open or closed). A Sentinel-2 image acquired on 07 December 2019 shows blockage of drainage conduits and partial refilling of Shispare Lake, which could pose a renewed secondary flood risk (Supplementary Fig. S10). However, cessation of surging and infill by icebergs may result in a smaller lake and smaller flood. The drainage on 22–23 June 2019 was well contained in the channel banks and resulted in little reported damage other than to a section of the Karakoram Highway. Nonetheless, the ice dam geometry and site of the drained lake should be monitored using satellite images and field observations. A high-resolution DEM would be valuable for discerning any future lake volume and for quantifying the susceptibility of the downstream communities to flood events.

The peak surge velocities of Shispare Glacier (~18 m d^−1^) were six-fold greater than Kyagar Glacier (~3 m d^−1^). Shispare Glacier has steep surface slopes and descends from a high elevation (7611–2567 m, a span of 5044 m in 16 km). Conversely, Kyagar Glacier descends from 7165 m to 4753 m (2412 m in 18 km). Shispare Glacier has more than twice the gradient (315 m km^−1^) of Kyagar Glacier (134 m km^−1^). Both glaciers are predominantly avalanche nourished, but Shispare Glacier receives large ice and snow avalanches from steep icefalls. The very high gravitational potential energy gradient drives rock and ice masses far down the glacier from high elevations.

Recently, Rashid *et al*.^35^ investigated the characteristics of the recent surge of Shispare Glacier using only Landsat 8 OLI data. This study reported that the surface flow maximum was 48 m d^−1^ during February–March 2019. Conversely, we observed ~18 ± 0.3 m d^−1^ surface flow maximum in June 2018 and May 2019. We are unsure how they obtained such high speeds, as our study using a higher temporal frequency of images did not find such high speeds using COSI-Corr and CIAS software. The higher maximum flow velocity values by Rashid *et al*.^35^ might be attributed to their utilization of Landsat 8 OLI images with cloud and snow conditions (see example Fig. 5 in cited reference). We observed that Landsat 8 OLI, ASTER and Sentinel-2 images with cloud, shadow and snow conditions failed to detect actual flow velocities^42,54^.

The surge peak of Shispare Glacier observed at the beginning of June 2018 (~18 ± 0.5 m d^−1^) involved the highest flow rate ever reported in the Karakoram using feature tracking (Supplementary Fig. S11). Other maximum reported velocities are for Hispar (~14 m d^−1^), Khurdopin (~15 m d^−1^) and Staghar (~10 m d^−1^) glaciers^20,23,24,27^ (Supplementary Fig. S11). However, the Hispar and Khurdopin had their highest surge velocity in summer from May through June 2015 and 2017 respectively. Staghar surged in the winter months during 1989. The exact reason for variability in surface velocity is unknown but largely influenced by topography (e.g., gradient), contribution of meltwater and ice mass.

Climate change and extreme weather probably affect surge dynamics through the intensification, timing, and elevation reach of avalanches, melting, crevassing, rock falls, and changes in glacier size. Threshold-triggered responses of surge-type glaciers probably do not correlate linearly to snowfall anomalies, thermal changes, or other conditioning or triggering factors, as the surge processes are numerous and complex. The ‘movement heterogeneity’ now established^20,24,26^ for these glaciers, contrasts with classic surge notions of short-lived fast flow and long-term stagnation and of sharply different processes in the reservoir and receiving zones. It is not yet clear whether movement heterogeneity closely connects to recent climatic and extreme weather disturbances, or might just now be better resolved due to improved satellite imaging and the advent of new analysis tools. The theoretical connections of surging to climate are strong, but the empirical evidence–aside from links to meltwater—is unclear. This may be due to additional factors that are not directly connected to climate, such as differing bed lithology (which affects subglacial till rheology and bed erosion, roughness, and shear resistance, and glacier sliding) and subglacial topography and glacier hypsometry (which affect the ability of the glacier to retain water and therefore shear stress and sliding). Each glacier’s small documented numbers of surges and uniqueness suggest that links to climate change and extreme weather may be most compellingly seen in the statistical regional behavior of surges, guided by case studies such as this. These are urgent matters for on-going research.

Overall, our study presents the surge dynamics of Shispare Glacier using feature tracking, DEM differencing, and thermal data to infer the seasonality of surface. We observed considerable heterogeneity of movement, including minor accelerations associated with summer ablation during the quiescent phase. According to local reports, two hydro-power power houses have closed, and a portion of an under-construction powerhouse was also affected by the glacier surge^5^. During 22–23 June 2019, the small GLOF damaged some part of the Karakoram Highway^55^. Muchuhar Glacier has also advanced~120 ± 47m during 2013–2019, which if surges again, could join the Shispare Glacier, and severely affect the downstream areas and potentially cause the disruptions of commerce along the Karakoram Highway. Therefore, a regular monitoring of the ice dam and Shispare Lake formation is suggested for potential GLOF warnings and to detect a likely surge of the Muchuhar tributary.

## Methods

### Satellite data and DEMs

We acquired 118 Landsat TM, ETM+, OLI and Sentinel-2 images, covering 1990 to 2019, with minimum clouds and the Shuttle Radar Topography Mission (SRTM) DEM from the USGS website (https://earthexplorer.usgs.gov/) (Supplementary Table S2 and S3). The surface displacement of Shispare Glacier from 2004 to 2011 were computed using ASTER images as Landsat 7 ETM + scenes are affected by scan line errors^56^. The ASTER product for DEM generation (AST_L1A) was obtained from the NASA EARTH SCIENCE DATA website (https://search.earthdata.nasa.gov/) under the flagship of the Global Land Ice Measurement from Space program^57^. The TanDEM-X DEM (90 m) was acquired from the German Aerospace Center (DLR) (https://geoservice.dlr.de/web/dataguide/tdm90/). Three Hexagon satellite images from 04 August 1973 (Images: DZB1206–500082L019001, DZB1206-500082L020001, and DZB1206-500082L021001) were obtained from USGS (https://earthexplorer.usgs.gov/) and used for DEM generation. Landsat, ASTER, and Sentinel-2 images were mainly used to compute surface displacements and surface melt elevations. The glacier outline was acquired from the Randolph Glacier Inventory (RGI 6.0)^58^ in a polygon shapefile format. We manually edited this glacier outline to improve the delineated boundary using a 10 m resolution Sentinel-2 image from 2019 to extract the surface displacement, elevation changes, and surface melt elevations. Eleven PlanetScope images^59^ were used to derive the lake area change and two were used to observe pre- and post-flood channel change using a normalized difference vegetation index (NDVI).

### Surface displacements and frontal change

A normalized cross-correlation (NCC) and phase correlation algorithms were used to derive multi-temporal surface flow velocity from two successive pairs of Landsat, ASTER or Sentinel-2 images using Image correlation software (CIAS)^42,60^ and COSI-Corr^43^, respectively. In CIAS, we used search window size ranged from 80 × 80 to 120 × 120 and a reference windows size of 10 × 10 and eliminated ≤0.6 correlation coefficient from the glacier flow data^20,60^. In COSI-Corr, we used a search window of 64×64 to 16×16 at a step size of 4 pixels. Directional filtering was also used to remove incorrect displacements. Finally, velocity vectors were visually verified on satellite images and any remaining erroneous displacements were removed. All the corrected surface displacements were converted to a daily scale.

The terminus of a surge glacier can be irregular. Therefore, frontal change was estimated using a glacier length tool developed for ArcGIS^61^. As this tool only uses the terminus of the glacier as input, we digitized these (not the entire glacier boundary) from the Landsat and Sentinel-2 satellite images.

### DEMs generation and elevation changes

We generated 14 ASTER DEMs using the orbital ancillary data by open-source Ames Stereo Pipeline (ASP) without any ground control points (GCPs) as suggested by Brun *et al*.^62^ and Shean *et al*.^63^ (Supplementary Table 4). Numerous studies have investigated the geometric evolution of surge-type glaciers using ASTER DEMs^24,44^. Additionally, we generated a 1973 DEM using three KH-9 Hexagon stereo images (04 August 1973) following a structure from motion with multi-view stereo workflow in AgisoftMetashape 1.5.3. We followed a similar method to Sevara^64^, which involved aligning the images using their fiducial marks, generating and filtering a sparse point cloud, georeferencing the model using 10 GCPs from Google Earth (root mean square error of 16 m)^65^, and producing a dense point cloud, which was used to generate a DEM. We used ‘high’ quality settings and input an image pixel size of 0.007 mm with a focal length of 304.8 mm.

The correction of systematic biases in elevation difference maps due to planimetric shift is essential for accurate results^66–68^. The coregistration of multiple DEMs is crucial to confirm corresponding pixels in the two DEMs correspond to the same ground position. Many studies have utilized SRTM data as a reference DEM to generate elevation difference maps and mass balance assessment for the surge glaciers^22,44,69,70^. We coregistered 1 Hexagon and 14 ASTER DEMs to the reference SRTM DEM on stable terrain with slopes <45° using the universal co-registration approach proposed by Nuth and Kääb^68^. A maximum of five iterations were performed to adjust all ASTER and Hexagon DEMs with the reference SRTM DEM so that horizontal shifts <1 m could be achieved^71^. The planimetric adjusted ASTER and Hexagon DEMs were subtracted from the reference SRTM DEM to produce elevation change maps. We excluded the outlier elevation-change ranges above the +150 and −150 m and the +200 and −200 m for ASTER and Hexagon DEMs, respectively.

### Estimation of surface temperature and surface melt elevations

We used 42 Landsat 8 Thermal Infrared Sensor (TIRS) brightness temperature images (2013–2019) and seven ASTER Surface Kinetic Temperature (AST_08) images (2001–2015) to derive the monthly glacier ‘melt elevation’ (−1.5°C < T < 1.5°C) (Supplementary Table 5). The brightness temperatures (Tb) from the Landsat TIRS imagery (band 10) were corrected for emissivity using the ASTER Global Emissivity Dataset and converted to surface temperature (S_t_)^72–74^

1 $${S}_{t}=\frac{{T}_{b}}{1+\left(\lambda \times \frac{{T}_{b}}{\rho }\right)ln\varepsilon }$$

where λ is wavelength of emitted radiance (band 10 peak response λ = 10.8 μm), ρ = 1.438×10^−2^ m K, and ε is the pixel emissivity. The Landsat data are not atmospherically corrected, in contrast to the ASTER data, but they are in agreement with the ASTER data. The mean and one standard deviation of surface melt elevations were extracted using the 30 m SRTM DEM^75^. The temperature range of the melt elevations accounts for measurement errors and actual temperature variations near the melting point due to local (subpixel) slopes and variable partial debris cover.

For a Standard Atmosphere semi-moist adiabatic lapse rate of 6.49**°** Celsius/km^76^, the 500 m difference between ASTER’s and Landsat’s melting elevations would imply roughly 3.2**°** Celsius difference, much more than recognized measurement errors. ASTER commonly reports temperatures that are higher than Landsat 8’s by about 0.8**°**^77^ Celsius. The acquisition times were 10:54 am and 10:40 am local time for ASTER and Landsat, respectively; ASTER’s 14 minutes later time of day might account for around 0.4**°** of the difference. Hence, 1.2**°** Celsius of the temperature and 200 m elevation difference in surface melt elevation is accounted for, but the rest might be due to weather. Likewise, in Fig. 6, the scatter may be partly or entirely due to weather if seasonal effects are removed. However, we recommend that the ASTER and Landsat teams undertake systematic cross comparison of temperature measurements over time, in case instrument drift is affecting measurements.

### Ice-dammed lake area, volume and drainage

Planet Scope imagery delivered at 3 m resolution after orthorectification and processing of surface reflectance^59^ was used to derive the ice-dammed Shispare Lake area change. The lake extent was manually digitized in each image due to the presence of floating ice, which would complicate water delineation based on band ratio techniques. For each time period, the mean lake level elevation was extracted from the 90 m TanDEM-X DEM (resampled to 5 m using bilinear interpolation to reduce edge effects) using the lake polygon boundary and excluding areas in contact with Shispare Glacier. The lake volume was derived for each lake polygon and one standard deviation of the lake level elevation was used to represent the lake level uncertainty.

Lake drainage was reported to have occurred on 22–23 June 2019^4^, which was confirmed using PlanetScope images from 9 June 2019 and 11 July 2019^59^. We derived a normalized difference vegetation index for 9 June 2019 using the near infrared and red bands of the PlanetScope images: NDVI values > =0.2 were classified as vegetation. The ALOS World 3D – 30 m DEM (AW3D30) was used to derive the area of vegetation in vertical elevation bands from the river channel. We visually compared false color composites (NIR, Red, Blue) of both PlanetScope images to identify areas of vegetation change.

### Uncertainty estimation

Since satellite data were utilized on different temporal and spatial resolutions therefore it is crucial to understand accuracy of results. We estimated uncertainty in glacier displacements proposed by previous studies^24,26^.

2 $$\sigma =365\frac{({C}_{\text{pix}}+\,{C}_{\text{match}})\Delta x}{\Delta t}$$

where C_pix_ is the uncertainty in co-registration in pixels (p), C_match_ is the uncertainty in the matching algorithm in pixels (p), Δx is the image resolution in meters, and Δ*t* is the time interval between the image pair in days. We used 0.5 p values for C_pix_ and C_match_ as proposed by Quincey *et al*.^26^ and Bhambri *et al*.^20^ (Supplementary Tables 2 and 3; Supplementary Fig. S12). The uncertainty in frontal change was estimated using the following equation proposed by Hall *et al*.^78^.

3 $$e=\sqrt{{(x1)}^{2}+{(x2)}^{2}}+{E}_{reg}$$

where;

e = error in frontal change,

x1 = pixel resolution of imagery 1,

x2 = pixel resolution of imagery 2,

E_reg_ = horizontal shift

We used the standard deviation of stable terrain (off glacier) as a standard statistical estimator for assessment of the uncertainty of surface elevation change^44^ (Supplementary Table 4; Supplementary Fig. S12).

## Supplementary information

Supplementary Information.
