# Supplementary material for: The hazardous 2017–2019 surge and river damming by Shispare Glacier, Karakoram
Source: Sci Rep. 2020 Mar 13;10:4685. doi: 10.1038/s41598-020-61277-8 (PMC7070009; doi:10.1038/s41598-020-61277-8)
Supplement: Supplementary file 1 — Supplementary Information. [file 41598_2020_61277_MOESM1_ESM.pdf]

Supplementary Information

The hazardous 2017-2019 surge and river damming by Shispare Glacier, Karakoram

Rakesh Bhambri[1], C. Scott Watson[2, 3], Kenneth Hewitt[4], Umesh K. Haritashya[5]*, Jeffrey S. Kargel[6], Arjun Pratap Shahi[7], Pritam Chand[8], Amit Kumar[1], Akshaya Verma[1] and Himanshu Govil[7]

[1] {Centre for Glaciology, Wadia Institute of Himalayan Geology, 33 GMS Road, Dehradun-248001, India}

[2] {COMET, School of Earth and Environment, University of Leeds, UK}

[3] {Department of Hydrology & Atmospheric Sciences, University of Arizona, USA}

[4] {Department of Geography and Environmental Studies, Wilfrid Laurier University, Waterloo, Canada}

[5] {Department of Geology, University of Dayton, 300 College Park, Dayton, OH 45469 USA}

[6] {Planetary Science Institute, Tucson, AZ 85719, USA}

[7] {Department of Applied Geology, National Institute of Technology, Raipur, Chhattisgarh, 492010, India}

[8] {Department of Geography, Central University of Punjab, Bathinda, Punjab, India, 151001}

*****Corresponding author: Umesh Haritashya (uharitashya1@udayton.edu)

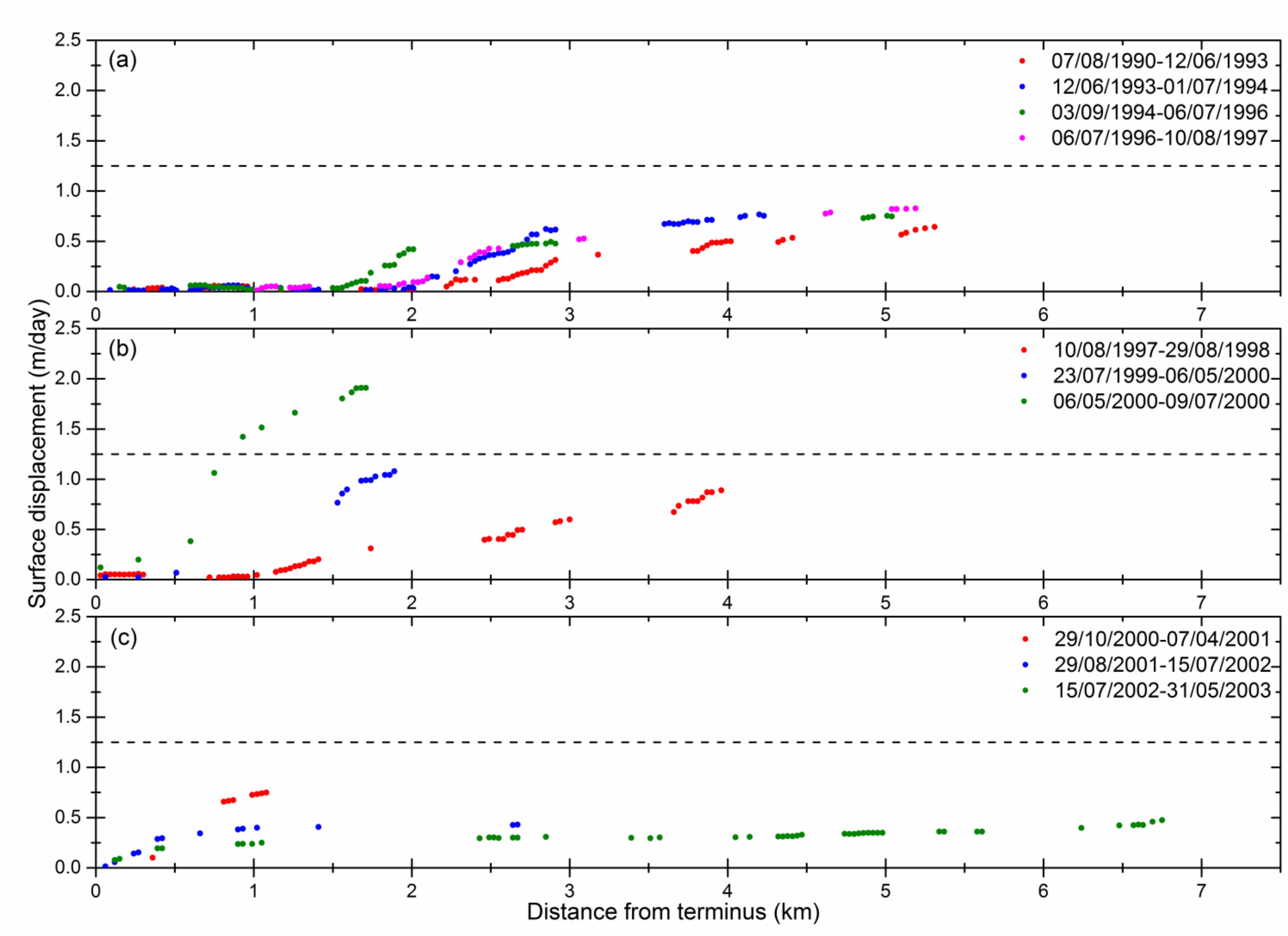

Supplementary Fig. S1 CIAS-derived surface displacement of Shispare Glacier during 1990-2003.

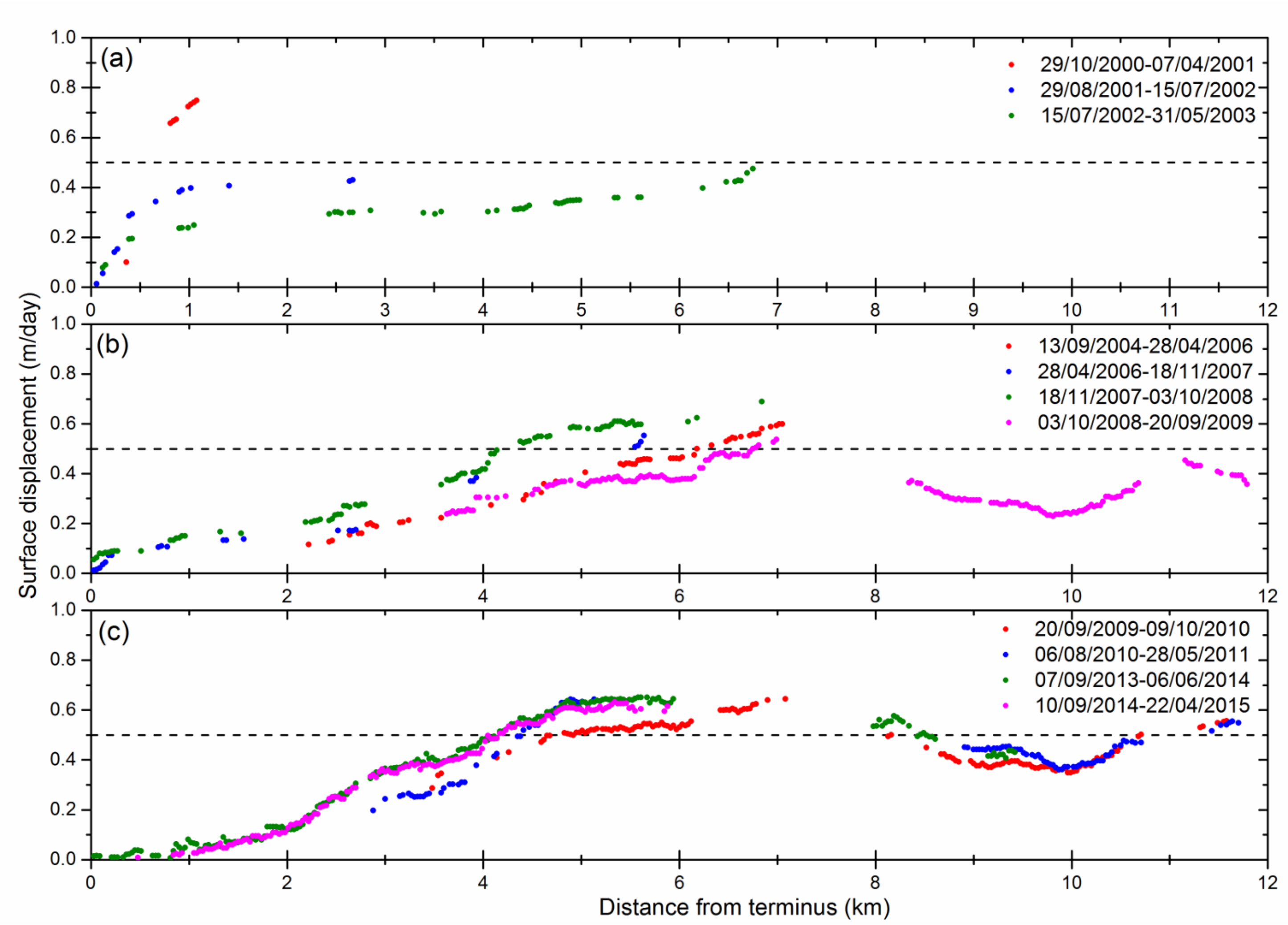

Supplementary Fig. S2 CIAS-derived surface displacement of Shispare Glacier during 2000-2015.

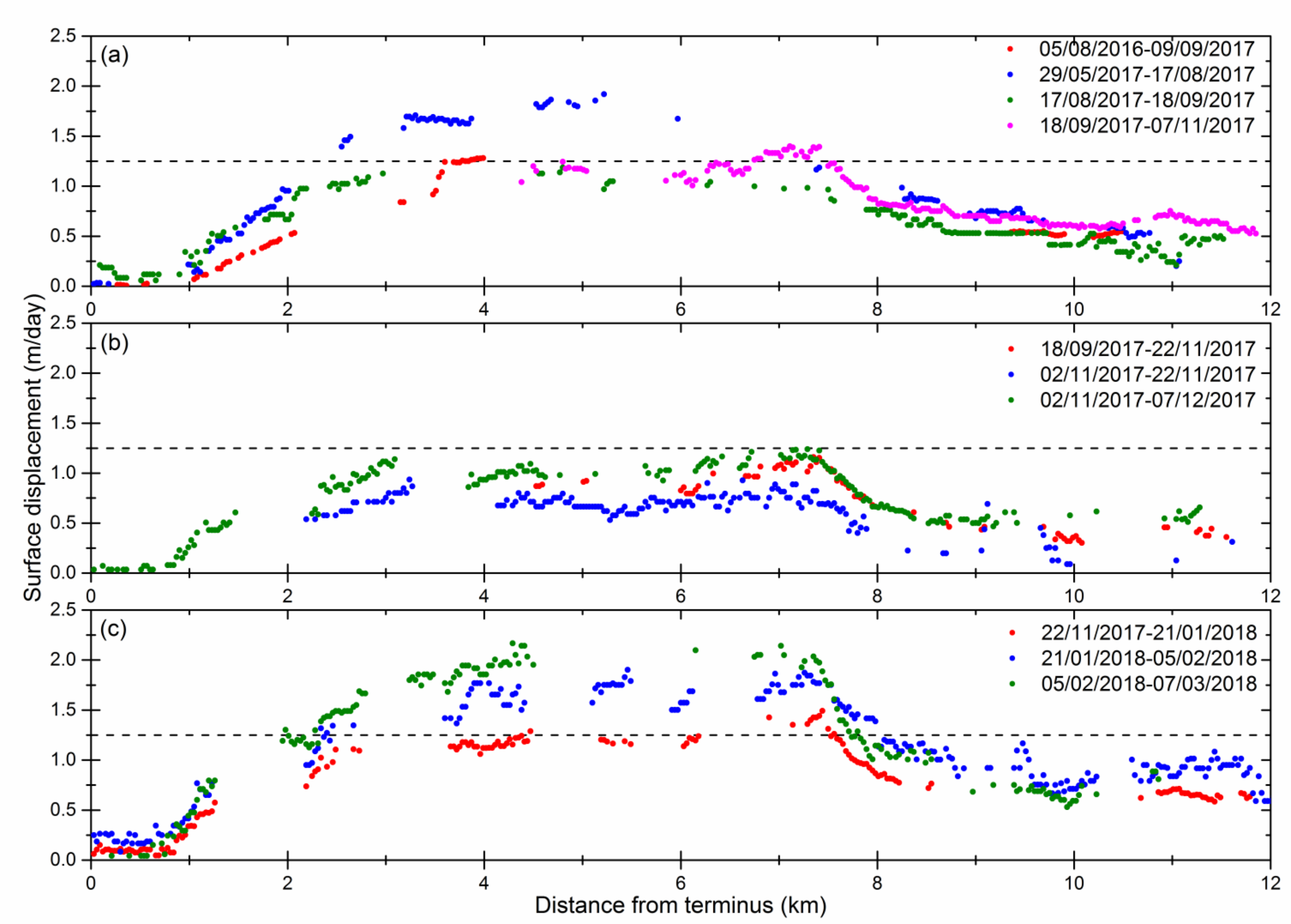

Supplementary Fig. S3 CIAS-derived surface displacement of Shispare Glacier during 2016-2018.

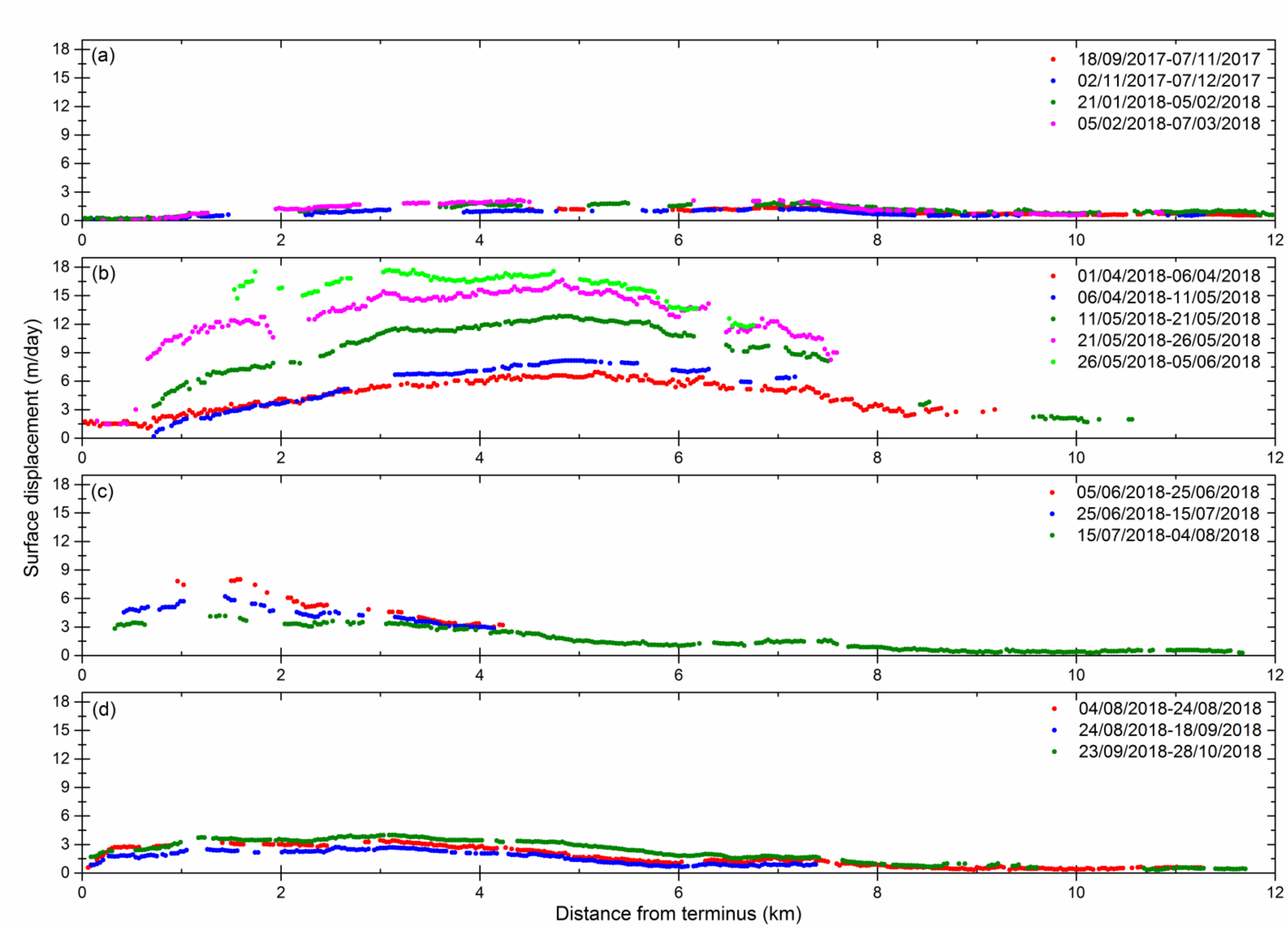

Supplementary Fig. S4 CIAS-derived surface displacement of Shispare Glacier during 2017-2018.

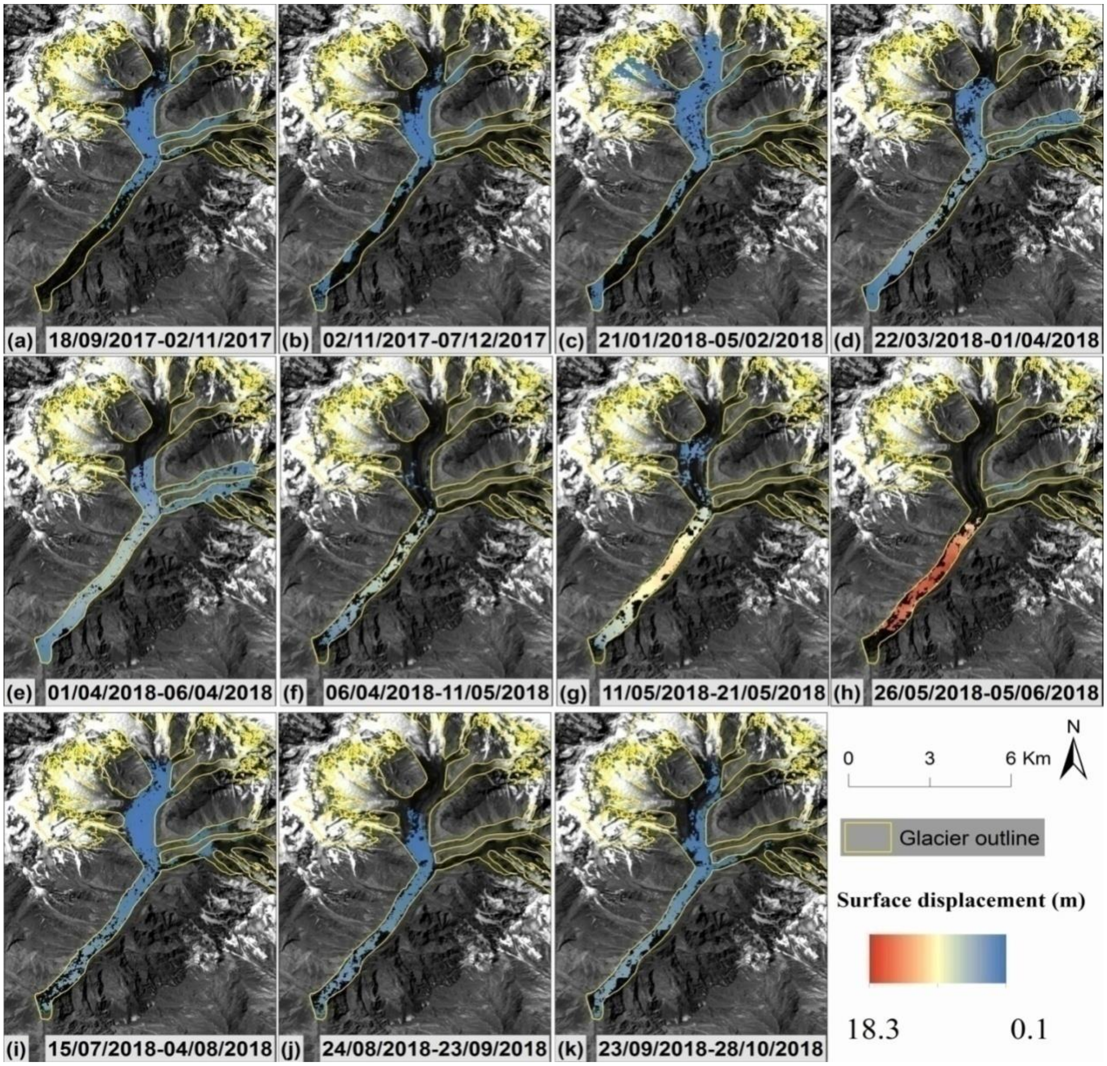

Supplementary Fig. S5 Surface displacement of Hasanabad Glacier during 2017-2018.

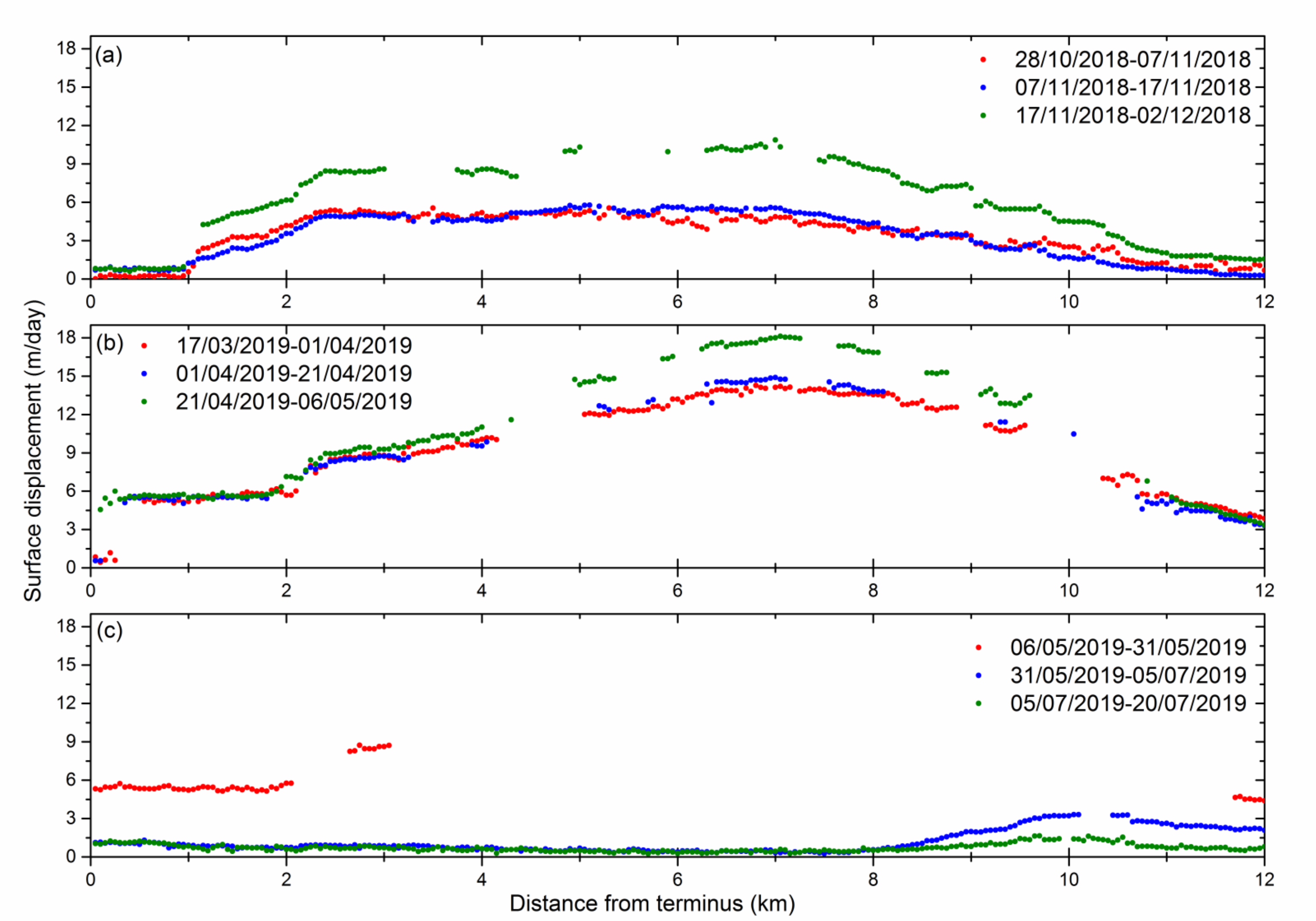

Supplementary Fig. S6 COSI-Corr-derived surface displacement of Shispare Glacier during 2018-2019.

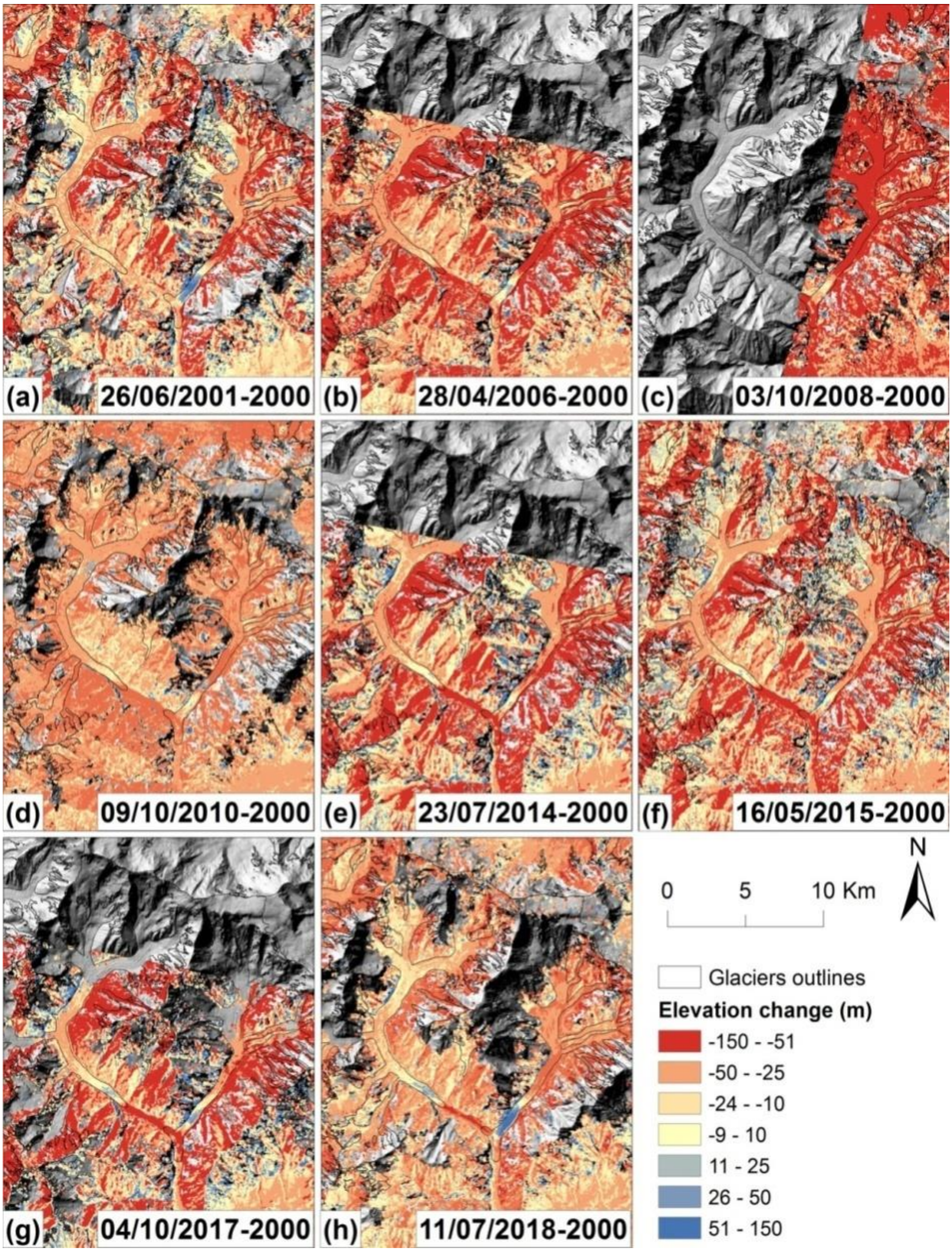

Supplementary Fig. S7 Elevation change of Hasanabad Glacier (Shispare and Muchuhar) using multiple ASTER and SRTM DEMs before the coregistration.

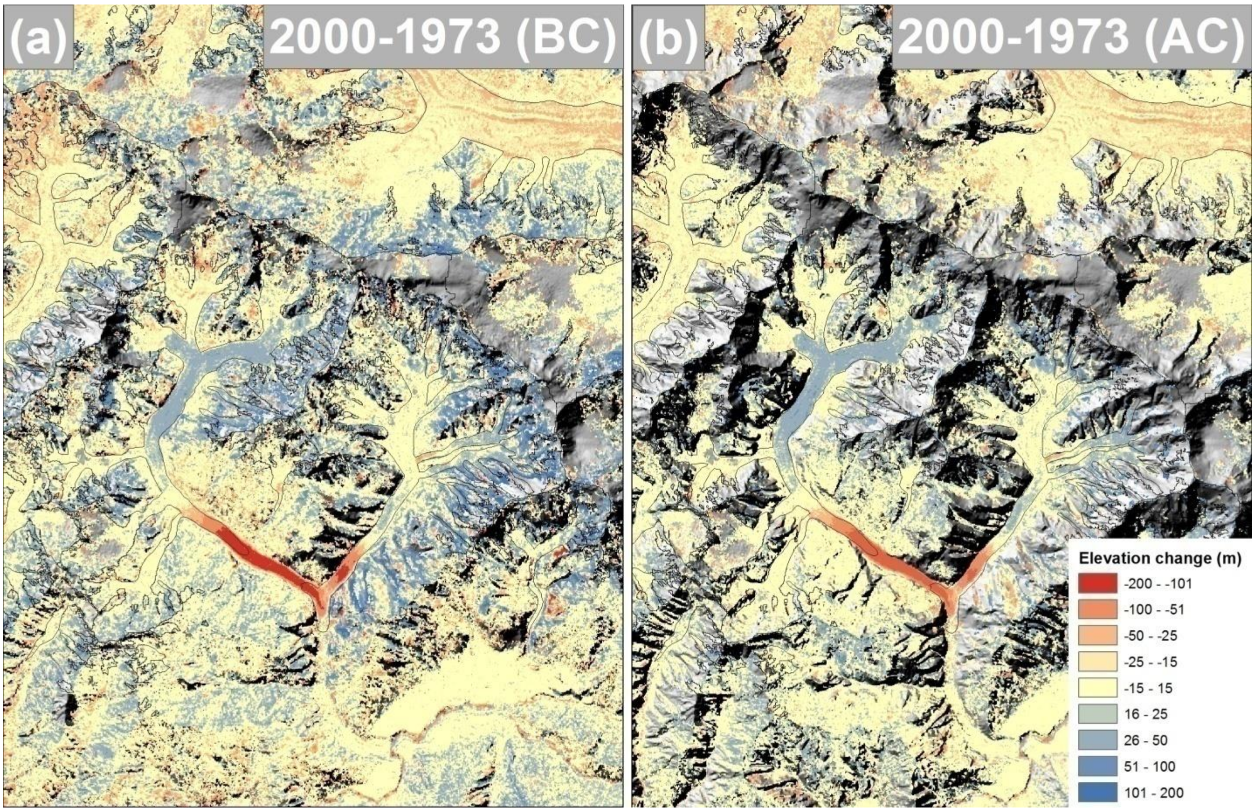

Supplementary Fig. S8 Elevation change of Hasanabad Glacier (Shispare and Muchuhar) using KH Hexagon and SRTM DEMs. BC and AC represents before (a) and after (b) the coregistration.

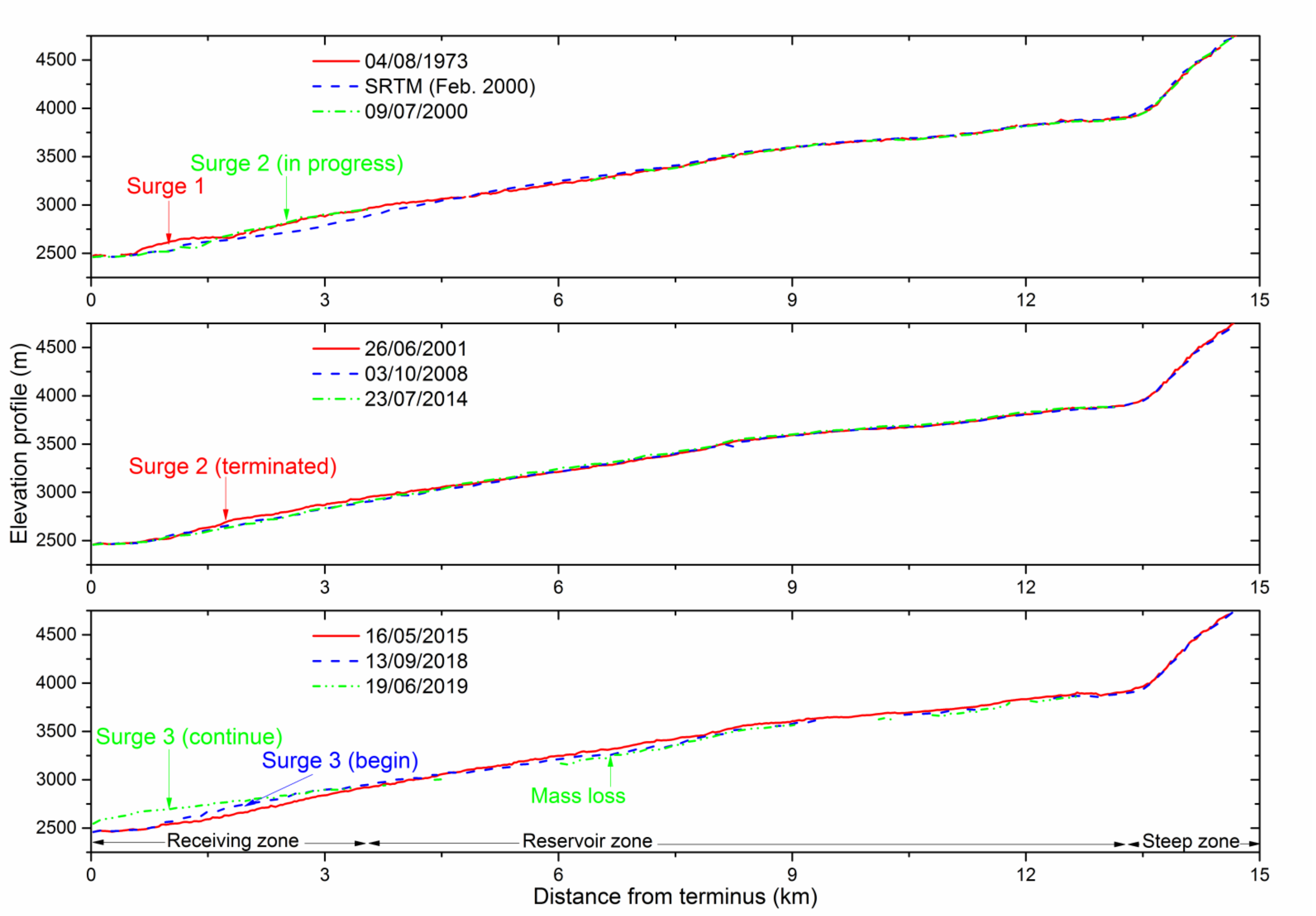

Supplementary Fig. S9 Elevation profile of Shispare Glacier using KH Hexagon, SRTM DEM, and multiple ASTER DEMs after the coregistration.

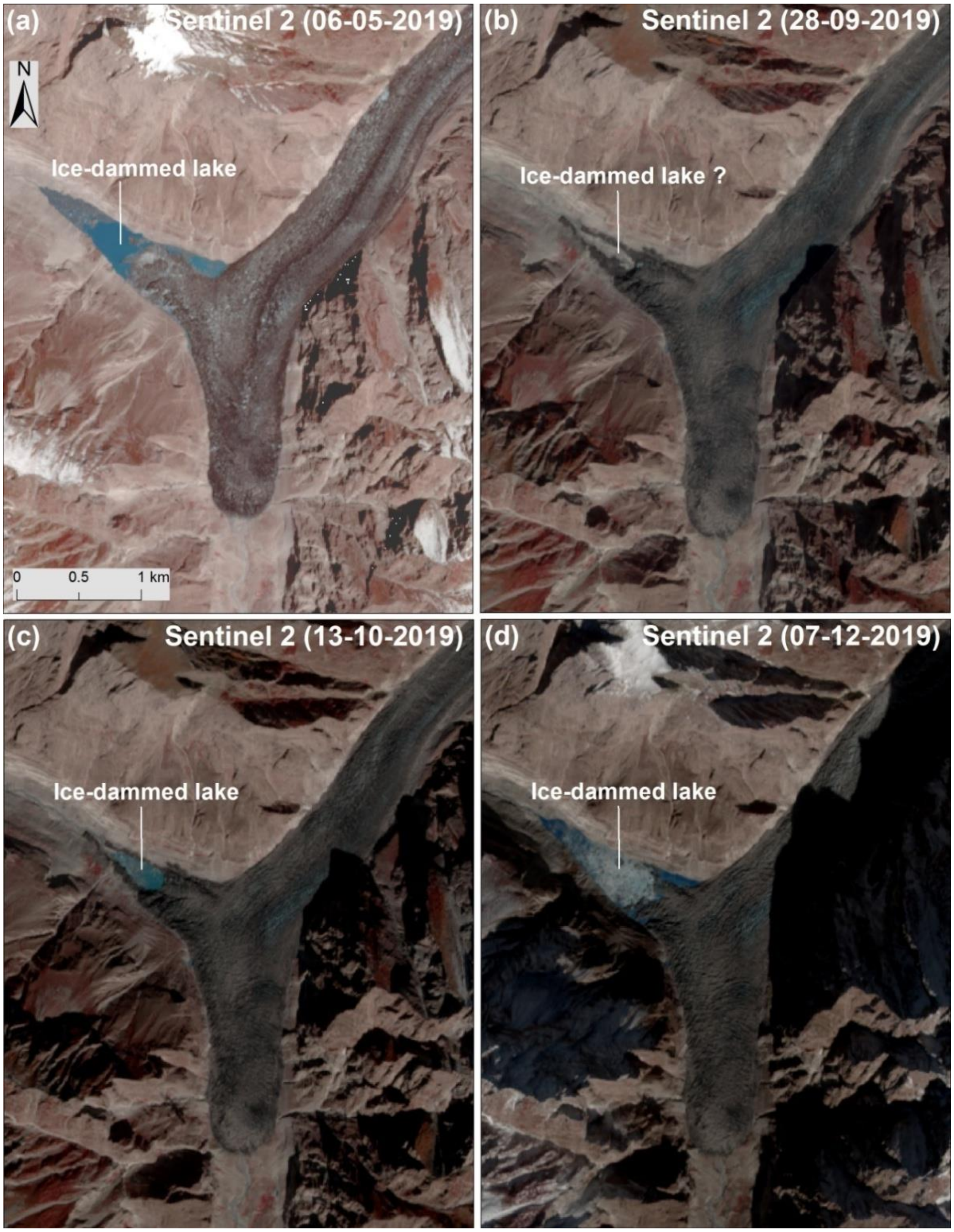

Supplementary Fig. S10 Fluctuation of Ice-dammed Shispare Lake. This lake (a) drained on 22–23 June 2019, started to fill (0.06 km^2) again in October 2019 (b, c), and increased its area (0.29 km^2) in December 2019 (d).

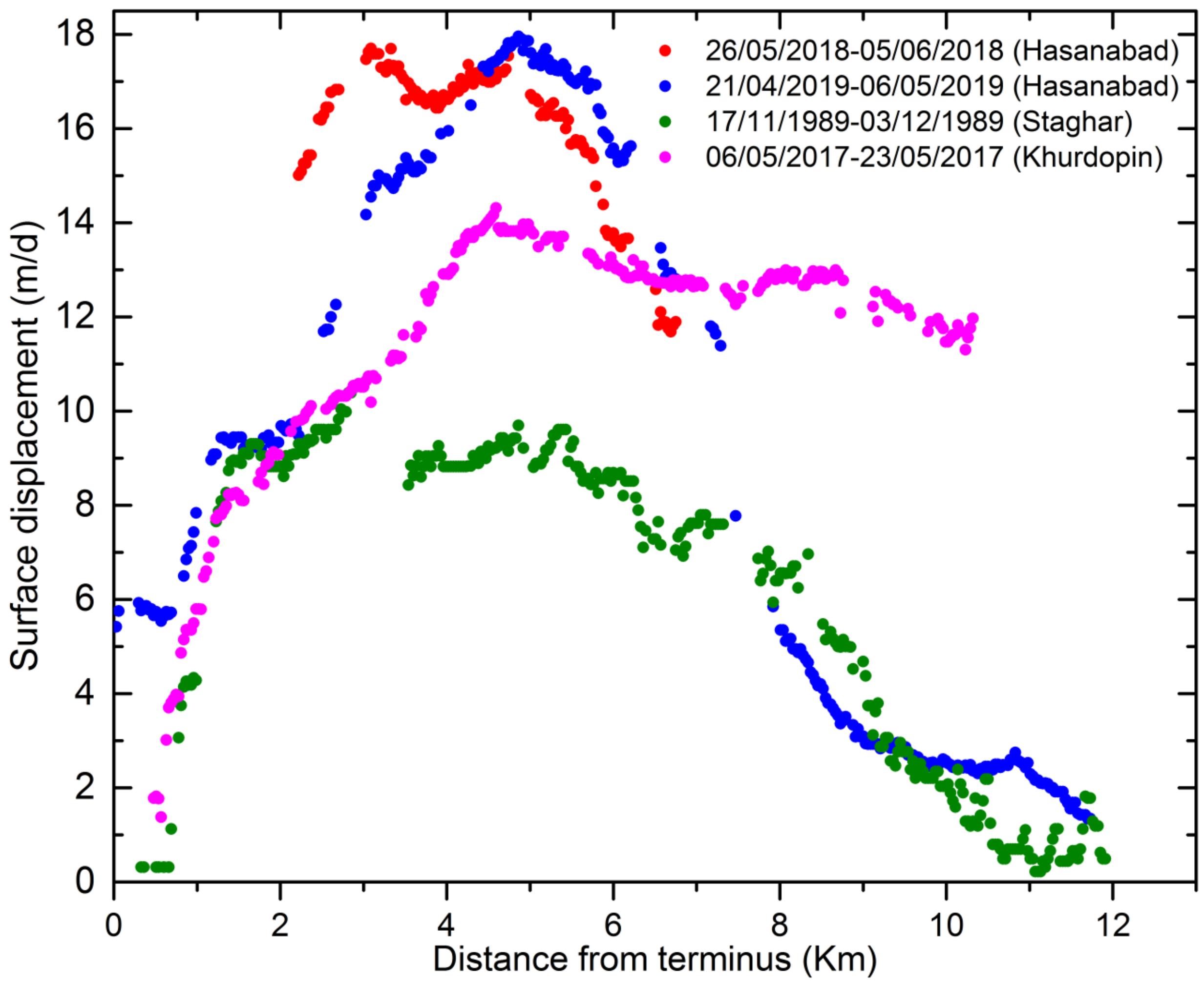

Supplementary Fig. S11 Comparison of peak surge displacement of glaciers in the Karakoram. Surface displacement of Shispare is computed in the present study whereas surface displacement of Staghar and Khurdopin is acquired from the previous studies (Bhambri et al. 2017; 2019)[1,2].

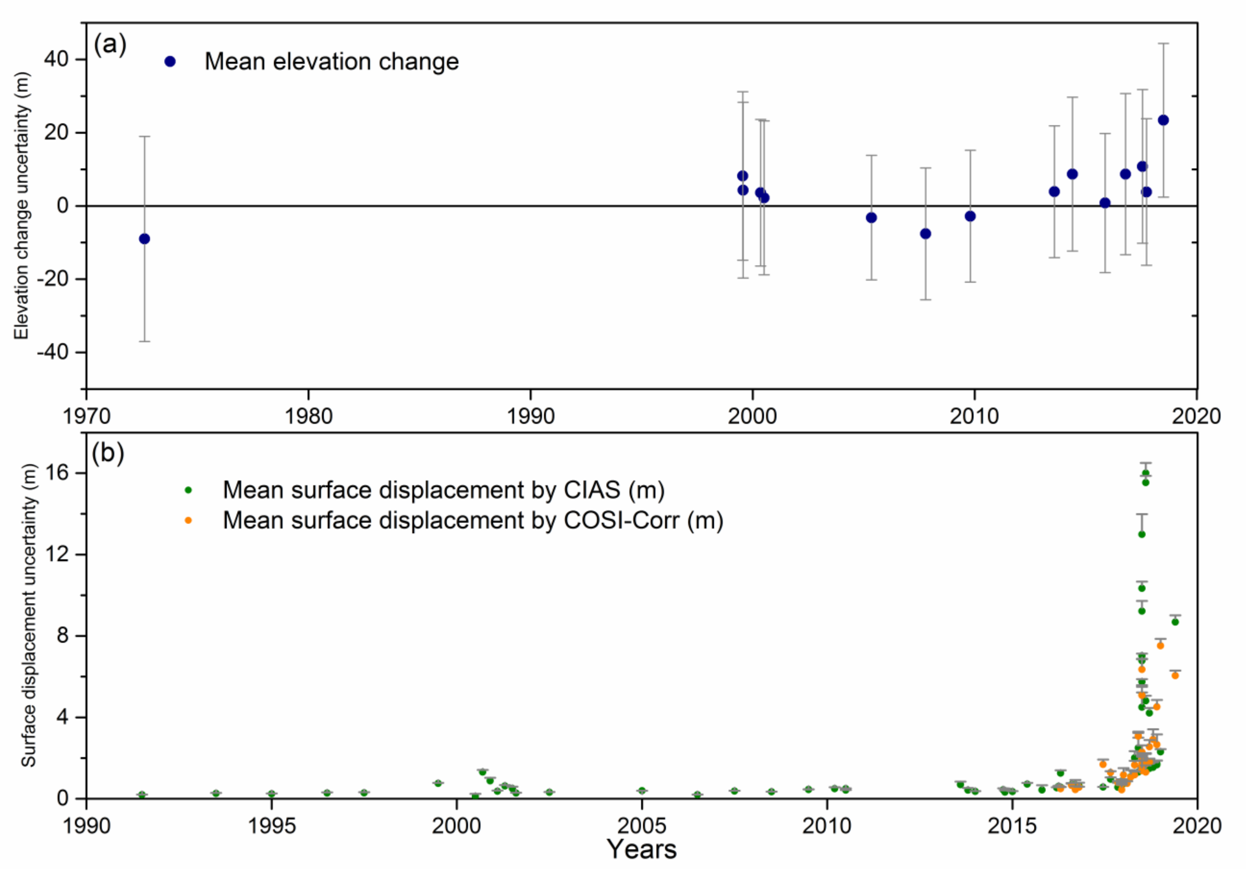

Supplementary Fig. S12 Uncertainty assessment of elevation change (a) and surface displacement (b) (Source: Supplementary Table 2, 3, 4).

Supplementary Table S1. Observations on Hasanabad Glacier by previous and present studies (modified from Goudie et al. 1984[3]).

Sl No.	Observer	Date	Details of Snout.	References
1	Ahmad Ali Khan	1889	9.65 km from Hasanabad Ravine Bridge (equivalent to 11.15 km from Hunza).	Mason (1930)[4]
2	Conway	1892	Roughly 14.3 km from Hunza.	Conway (1894)[5]
3	Abdul Gaffar	1893	Snout 4.93 km from Hunza.	Mason (1930)[4]
4	Neve	1895	Reports local comments that ice had advanced 3.2 km that year and 6.4 to 8 km in previous year. This probably refers to movements of 1892/1893.	Mason (1930)[4]
5	Hayden	1903	Told that glacier had advanced c. 9.7 km ("a day's march") in two and half months.	Hayden (1907)[6]
6	Hayden	1906	Same as 1903. Surveyed.	Hayden (1907)[6]
7	Workmans	1908	Same as 1906.	Workman and Workman (1910)[7]

8	Mason	1913	Same as 1906 (3.3 km from Hunza)	Mason (1914)[8]
9	Visser	1925	Volume of ice decreased. Portion of snout was dead ice. Length the same as 1913.	Visser (1928)[9]
10	Todd	1929	Glacier dirty. Snout 2.4 km from Ravine Bridge and 366 m upstream from Hayden's marks.	Mason (1930)[4]
11	Mason	1932	c. 550 m back from 1906 maximum (3879 m from Hunza).	Mason (1935)[10]
12	Paffen et al.	1954	Glacier had split into its two tributary valley glaciers. Muchuhar at 11790 m from Hunza, and Shispare 9286 m from Hunza (equivalent to 7 km and 4.5 km retreat since 1929).	Paffen et al. (1956)[11]
13	Zhang Xiangsong	1979	Glaciers re-united and just beyond confluence. Muchuhar 6990 m from Hunza and Shispare 7786 m.	Batura Glacier Investigation Group (1976)[12]
14	International Karakoram Project	1980	Nearest point of snout to Hunza = 7200 m.	Goudie et al. (1984)[3]
15	Bhambri et al.	2017	Muchuhar and Shispare meet both tributaries before 1972 due to advance of Muchuhar. Shispare surged during 1972-76 and again from	Bhambri et al. (2017)[1]

			1993 to 2002. However, Muchuhar could not meet with Shispare due to retreat of ~4 km.	
16	Bhambri et al.	2019	Shispare Glacier's terminus advanced 1495 ± 47 m between 2017 and 2019.	Present study

Supplementary Table S2. Details for satellite image pairs used for automated surge glacier surface feature tracking in CIAS with the estimated uncertainty.

**S.No.**	**Satellite image pairs dates matched**	**Temporal separation (days)**	**Sensors**	**Pixel resolution (m)**	**Estimated uncertainty (m/day)**
1	07/08/1990-12/06/1993	1040	TM-TM	30	0.01
2	12/06/1993-01/07/1994	384	TM-TM	30	0.03
3	03/09/1994-06/07/1996	672	TM-TM	30	0.02
4	06/07/1996-10/08/1997	400	TM-TM	30	0.03
5	10/08/1997-29/08/1998	384	TM-TM	30	0.03
6	23/07/1999-06/05/2000	288	ETM+-ETM+	15	0.03
7	06/05/2000-09/07/2000	64	ETM+-ETM+	15	0.12
8	09/07/2000-11/09/2000	64	ETM+-ETM+	15	0.11
9	11/09/2000-29/10/2000	48	ETM+-ETM+	15	0.15
10	11/09/2000-07/04/2001	208	ETM+-ETM+	15	0.03
11	29/10/2000-07/04/2001	160	ETM+-ETM+	15	0.04
12	07/04/2001-26/06/2001	80	ETM+-ETM+	15	0.09
13	29/08/2001-15/07/2002	320	ETM+-ETM+	15	0.02
14	15/07/2002-31/05/2003	320	ETM+-ETM+	15	0.02
15	13/09/2004-28/04/2006	592	ASTER-ASTER	10	0.008
16	28/04/2006-18/11/2007	569	ASTER-ASTER	10	0.008

17	18/11/2007-03/10/2008	320	ASTER-ASTER	10	0.01
18	03/10/2008-20/09/2009	352	ASTER-ASTER	10	0.01
19	20/09/2009-06/08/2010	320	ASTER-ASTER	10	0.01
20	20/09/2009-09/10/2010	384	ASTER-ASTER	10	0.01
21	06/08/2010-09/10/2010	64	ASTER-ASTER	10	0.07
22	06/08/2010-28/05/2011	295	ASTER-ASTER	10	0.01
23	09/10/2010-28/05/2011	231	ASTER-ASTER	10	0.02
24	18/05/2013-05/07/2013	48	OLI-OLI	15	0.15
25	05/07/2013-07/09/2013	64	OLI-OLI	15	0.11
26	07/09/2013-06/06/2014	272	OLI-OLI	15	0.02
27	06/06/2014-10/09/2014	96	OLI-OLI	15	0.07
28	08/07/2014-10/09/2014	64	OLI-OLI	15	0.11
29	08/07/2014-26/09/2014	80	OLI-OLI	15	0.09
30	10/09/2014-22/04/2015	224	OLI-OLI	15	0.03
31	22/04/2015-12/08/2015	112	OLI-OLI	15	0.06
32	12/08/2015-13/09/2015	32	OLI-OLI	15	0.23
33	19/08/2015-01/05/2016	256	OLI-OLI	15	0.02
34	19/08/2015-18/06/2016	304	OLI-OLI	15	0.02
35	13/09/2015-10/05/2016	240	OLI-OLI	15	0.03
36	01/05/2016-18/06/2016	48	OLI-OLI	15	0.15
37	10/05/2016-01/10/2016	144	OLI-OLI	15	0.05

38	18/06/2016-20/07/2016	32	OLI-OLI	15	0.23
39	18/06/2016-05/08/2016	48	OLI-OLI	15	0.15
40	05/08/2016-09/09/2017	400	OLI-OLI	15	0.01
41	29/05/2017-17/08/2017	80	OLI-OLI	15	0.09
42	17/08/2017-18/09/2017	32	OLI-OLI	15	0.23
43	18/09/2017-07/11/2017	50	Sentinel2- Sentinel2	10	0.1
44	18/09/2017-22/11/2017	65	Sentinel2- Sentinel2	10	0.07
45	02/11/2017-22/11/2017	20	Sentinel2- Sentinel2	10	0.25
46	02/11/2017-07/12/2017	35	Sentinel2- Sentinel2	10	0.14
47	22/11/2017-21/01/2018	60	Sentinel2- Sentinel2	10	0.08
48	21/01/2018-05/02/2018	15	Sentinel2- Sentinel2	10	0.33
49	05/02/2018-07/03/2018	30	Sentinel2- Sentinel2	10	0.16
50	07/03/2018-22/03/2018	15	Sentinel2- Sentinel2	10	0.33
51	17/03/2018-01/04/2018	15	Sentinel2- Sentinel2	10	0.33
52	22/03/2018-01/04/2018	10	Sentinel2- Sentinel2	10	0.5
53	01/04/2018-06/04/2018	5	Sentinel2- Sentinel2	10	1
54	01/04/2018-11/05/2018	40	Sentinel2- Sentinel2	10	0.12
55	01/04/2018-21/05/2018	50	Sentinel2- Sentinel2	10	0.1
56	06/04/2018-11/05/2018	35	Sentinel2- Sentinel2	10	0.14
57	06/04/2018-21/05/2018	45	Sentinel2- Sentinel2	10	0.11
58	11/05/2018-21/05/2018	10	Sentinel2- Sentinel2	10	0.5

59	11/05/2018-26/05/2018	15	Sentinel2- Sentinel2	10	0.33
60	21/05/2018-26/05/2018	5	Sentinel2- Sentinel2	10	1
61	21/05/2018-05/06/2018	15	Sentinel2- Sentinel2	10	0.33
62	26/05/2018-05/06/2018	10	Sentinel2- Sentinel2	10	0.5
63	05/06/2018-25/06/2018	20	Sentinel2- Sentinel2	10	0.25
64	25/06/2018-15/07/2018	20	Sentinel2- Sentinel2	10	0.25
65	15/07/2018-04/08/2018	20	Sentinel2- Sentinel2	10	0.25
66	04/08/2018-24/08/2018	20	Sentinel2- Sentinel2	10	0.25
67	24/08/2018-18/09/2018	25	Sentinel2- Sentinel2	10	0.2
68	24/08/2018-23/09/2018	30	Sentinel2- Sentinel2	10	0.16
69	23/09/2018-28/10/2018	35	Sentinel2- Sentinel2	10	0.14
70	21/04/2019-06/05/2019	15	Sentinel2- Sentinel2	10	0.33

Supplementary Table S3. Details for Sentienl-2 (10 m spatial resolution) satellite image pairs used for automated surge glacier surface feature tracking in COSI-Corr with the estimated uncertainty.

S.No	**Satellite image pairs dates matched**	**Temporal separation (days)**	**Pixel resolution (m)**	**Estimated uncertainty (m/day)**
1	05/07/2019-20/07/2019	15	10	0.33
2	31/05/2019-05/07/2019	35	10	0.14
3	06/05/2019-31/05/2019	25	10	0.20
4	21/04/2019-06/05/2019	15	10	0.33
5	01/04/2019-21/04/2019	20	10	0.25
6	17/03/2019-01/04/2019	15	10	0.33
7	17/11/2018-02/12/2018	15	10	0.33
8	07/11/2018-17/11/2018	10	10	0.50
9	28/10/2018-07/11/2018	10	10	0.50
10	23/10/2018-07/11/2018	15	10	0.33
11	23/09/2018-23/10/2018	30	10	0.17
12	08/09/2018-23/09/2018	15	10	0.33
13	29/08/2018-08/09/2018	10	10	0.50

14	14/08/2018-29/08/2018	15	10	0.33
15	14/08/2018-24/08/2018	10	10	0.50
16	04/08/2018-14/08/2018	10	10	0.50
17	25/07/2018-04/08/2018	10	10	0.50
18	15/07/2018-25/07/2018	10	10	0.50
19	10/07/2018-25/07/2018	15	10	0.33
20	26/05/2018-10/07/2018	45	10	0.11
21	11/05/2018-21/05/2018	10	10	0.50
22	01/05/2018-11/05/2018	10	10	0.50
23	06/04/2018-01/05/2018	25	10	0.20
24	01/04/2018-01/05/2018	30	10	0.17
25	07/03/2018-01/04/2018	25	10	0.20
26	05/02/2018-07/03/2018	30	10	0.17
27	21/01/2018-05/02/2018	15	10	0.33
28	07/12/2017-21/01/2018	45	10	0.11
29	22/11/2017-07/12/2017	15	10	0.33
30	07/11/2017-22/11/2017	15	10	0.33
31	02/11/2017-22/11/2017	20	10	0.25

32	18/09/2017-02/11/2017	45	10	0.11
33	25/07/2017-18/09/2017	55	10	0.09
34	06/05/2017-25/07/2017	80	10	0.06
35	16/04/2017-06/05/2017	20	10	0.25
36	27/11/2016-16/04/2017	140	10	0.04
37	28/10/2016-27/11/2016	30	10	0.17
38	18/09/2016-28/10/2016	40	10	0.13
39	20/07/2016-18/09/2016	60	10	0.08
40	30/06/2016-20/07/2016	20	10	0.25
41	21/05/2016-30/06/2016	40	10	0.13
42	01/05/2016-21/05/2016	20	10	0.25

Supplementary Table S4. Uncertainty assessment of DEMs used in the study.

S.No.	Scene ID	Satellite type	Date	Shift X	Shift Y	Shift Z	$MEAN_{bef}$	STD_{bef}	$MEAN_{aft}$	STD_{aft}
1	AST_L1A_00306192019055906 _20190628024838_12262	ASTER	19-06-2019	8	-29	-43	-41	33	0.3	21
2	AST_L1A_00309132018055405 _20190609032457_3181	ASTER	13-09-2018	27	-26	-43	-47	36	-3	20
3	AST_L1A_00307112018055419 _20190609032447_3116	ASTER	11-07-2018	26	-21	-39	-43	35	-3	21
4	AST_L1A_00304102017055859 _20190609032457_3189	ASTER	04-10-2017	44	-38	-39	-45	43	-1	22
5	AST_L1A_00311012016055909 _20190609032507_3236	ASTER	01-11-2016	30	-37	-45	-44	37	-1	19
6	AST_L1A_00305162015055329 _20190609032507_3251	ASTER	16-05-2015	27	-36	-48	-46	38	-2	21

7	AST_L1A_00307232014055856 _20190609032507_3254	ASTER	23-07-2014	42	-47	-45	-45	45	-2	18
8	AST_L1A_00310092010055227 _20190609032517_3289	ASTER	09-10-2010	-15	-26	-45	-44	28	-3	18
9	AST_L1A_00310032008055305 _20190609032447_3131	ASTER	03-10-2008	-18	-24	-57	-56	26	-2	18
10	AST_L1A_00304282006055824 _20190609032447_3134	ASTER	28-04-2006	13	-31	-41	-43	31	-1	17
11	AST_L1A_00306262001060049 _20190609032457_3176	ASTER	26-06-2001	36	-19	-31	-32	38	-3	21
12	AST_L1A_00304302001060859 _20190628024838_12265	ASTER	30-04-2001	5	-25	-42	-43	29	-1	20
13	AST_L1A_00307162000061408 _20190628024838_12269	ASTER	16-07-2000	26	-33	-28	-27	40	-2	24
14	AST_L1A_00307092000060804	ASTER	9-07-2000	30	-9	-36	-40	35	-2	23

	_20190628024838_12271									
15	DZB1206-500082L019001	Hexagon	04-08-1973	1	-31	1	1	28	3	28

Supplementary Table S5. Thermal imagery used to derive the glacier melt elevations.

Thermal data	Date	Day of year	Mean melt (temperature > -1.5 <1.5°C) elevation (m)	Standard deviation
Landsat	10/9/2013	282	5992	604
	10/25/2013	298	4486	429
	6/6/2014	157	5689	395
	7/8/2014	189	5859	317
	7/24/2014	205	6356	353
	8/25/2014	237	5631	475
	9/10/2014	253	5016	285
	9/26/2014	269	5417	552
	3/21/2015	80	3988	264
	4/6/2015	96	3327	179
	4/22/2015	112	4442	383
	9/13/2015	256	5395	324
	10/31/2015	304	4902	606
	11/16/2015	320	3196	327
	5/10/2016	131	5536	282

	10/1/2016	275	5905	339
	11/2/2016	307	4468	381
	4/11/2017	101	4436	492
	5/13/2017	133	5509	667
	5/29/2017	149	6015	377
	8/1/2017	213	6305	450
	9/18/2017	261	5352	566
	4/14/2018	104	5014	475
	4/30/2018	120	5696	414
	8/4/2018	216	6334	502
	8/20/2018	232	6320	485
	3/16/2019	75	3526	364
	4/1/2019	91	4101	477
	4/10/2014	100	3920	252
	6/29/2014	180	5951	444
	7/15/2014	196	6165	415
	9/17/2014	260	5802	545
	10/3/2014	276	5451	540
	4/13/2015	103	5117	503
	8/19/2015	231	6220	546

	2/27/2016	58	4194	315
	5/1/2016	122	5418	460
	9/9/2017	252	6090	420
	3/20/2018	79	4010	371
	4/5/2018	95	5068	463
	6/8/2018	159	5908	381
	7/10/2018	191	6376	400
ASTER	8/6/2010	218	5996	855
	5/2/2010	122	4546	729
	5/16/2015	136	4969	740
	6/26/2001	177	5526	516
	7/11/2018	192	5885	937
	10/3/2008	277	5043	601
	10/9/2010	282	4435	359

References

1. Bhambri, R., Hewitt, K., Kawishwar, P. & Pratap, B. Surge-type and surge-modified glaciers in the Karakoram. *Sci. Rep.* **7**, 15391 (2017).
2. Bhambri, R. *et al.* Ice-dams, outburst floods, and movement heterogeneity of glaciers, Karakoram. *Glob. Planet. Change* **180**, 100–116 (2019).
3. Goudie, A. S., Jones, D. K. C. & Brunsden, D. Recent fluctuations in some glaciers of the Western Karakoram mountains, Hunza, Pakistan. in *The international Karakoram project* (ed. Miller, K. J.) **2**, 411–455 (1984).
4. Mason, K. The glaciers of the Karakoram and neighbourhood. *Rec. Geol. Surv. India* **63**, 214–278 (1930).
5. Conway, W. M. *Climbing and Exploration in the Karakoram Himalayas*. (T. Fisher Unwin, 1894).
6. Hayden, H. H. Notes on certain glaciers in Northwest Kashmir. *Rec. Geol. Surv. India* **35**, 127–137 (1907).
7. Workman, W. H. The Tongue of the Hasanabad Glacier in 1908. *Geogr. J.* **36**, 194–196 (1910).
8. Mason, K. Examination of Certain Glacier Snouts of Hunza and Nagar. *Rec. Geol. Surv. India* **6**, 49–51 (1914).
9. Visser, P. C. Von dem Gletschern am Oberstn Indus. *Zeitschrift Fur Glets.* **16**, 169–229 (1928).
10. Mason, K. The study of threatening glaciers. *Geogr. J.* **85**, 24–35 (1935).
11. Paffen, K. H., Pillewizer, W. & Schneider, H.-J. FORSCHUNGEN IM HUNZA-KARAKORUM: Vorläufiger Bericht über die wissenschaftlichen Arbeiten der Deutsch-

Österreichischen Himalaya-Karakorum-Expedition 1954. *Erdkunde* 1–33 (1956).

12. ‘Batura Investigations Group’. The Batura Glacier in the Karakoram Mountains and its variations. *Scientia Sinica* **22**, 958–974 (1979).
